# Facile One-Step Electrodeposition Preparation of Cationic Pillar[6]arene-Modified Graphene Films on Glassy Carbon Electrodes for Enhanced Electrochemical Performance

**DOI:** 10.3389/fchem.2020.00430

**Published:** 2020-06-04

**Authors:** Qunpeng Duan, Lijie Wang, Fei Wang, Hongsong Zhang, Kui Lu

**Affiliations:** ^1^School of Materials and Chemical Engineering, Henan University of Engineering, Zhengzhou, China; ^2^School of Chemical Engineering and Food Science, Zhengzhou Institute of Technology, Zhengzhou, China

**Keywords:** electrodeposition, pillar[6]arene, host-guest inclusion, graphene films, electrochemical performance

## Abstract

In the present work, we have developed a facile one-step route for preparing electrochemically reduced graphene oxide-cationic pillar[6]arene (ErGO-CP6) nanocomposite films on glassy carbon electrodes (GCEs) directly from graphene oxide-cationic pillar[6]arene (GO-CP6) colloidal solution by using a pulsed electrodeposition technique. The electrocatalytic activity of ErGO-CP6 was examined by studying the oxidations of five purine bases [adenine (A), guanine (G), xanthine (X), hypoxanthine (HX), and uric acid (UA)]. It enhanced the oxidation currents of A, G, X, HX, and UA when compared to unmodified ErGO films and bare GCE, which is considered to be the synergetic effects of the graphene (excellent electrical properties and large surface area) and CP6 molecules (high inclusion complexation and enrichment capability).

## Introduction

Graphene, a 2D sp^2^-hybirdized carbon sheet, has attracted considerable attention in academia and industry due to its fascinating electronic, chemical, mechanical, thermal, and optical properties as well as for its tremendous potential in applications in various fields, such as nanoelectronics (Son et al., 2006), supercapacitors (Maiti et al., 2014), batteries (Takamura et al., 2007), sensors (Shao et al., 2010b), and nanocomposites (Vickery et al., 2009). The reduced graphene oxide (rGO) is the product of treating graphene oxide (GO) under reducing conditions. Although rGO has a relatively lower conductivity than that of the graphene made with a mechanical cleaving method, it is nevertheless a versatile material. In particular, it can be used as a perfect candidate for carbon-based electrode materials to produce electrochemical sensors or biosensors owing to its large active surface area, good electrical conductivity, and electrocatalytic activity (Zhou et al., 2009). However, the practical applications of rGO are challenged by its irreversible agglomeration in an aqueous solution, which significantly reduces its effectiveness. Interestingly, introducing water-soluble macrocyclic hosts as functional molecules can effectively disperse graphene and further introduce new or enhanced functions through combining their individual characteristics. Therefore, macrocyclic-host-functionalized rGO nanocomposites that simultaneously possess the unique properties of rGO (a large surface area and good conductivity) and the macrocyclic host (high supramolecular recognition and good enrichment capability) have been intensively exploited as electrocatalysts for improving the analyte detection sensitivity (Guo et al., 2010, 2011; Xu et al., 2011; Zhou et al., 2013a,b,c; Li et al., 2017; Singh et al., 2018; Sun et al., 2019; Tan et al., 2019a). The commonly reported approach for the preparation of the macrocyclic-host-functionalized rGO nanocomposite modified electrode is the drop-casting of chemically reduced graphene oxide-macrocyclic host suspension onto the electrode surface. Obviously, such a preparation methodology involves highly toxic chemicals, such as hydrazine hydrate, and, moreover, chemical reduction of the graphene oxide-macrocyclic host suspension cannot completely reduce oxygen-containing functional groups, which may result in a decrease in the electrochemical performance.

More recently, electrochemical reduction of GO to rGO has attracted considerable attention because it is regarded as a simple, fast, and green method; in addition, graphene film can be obtained by this method on conductive substrates (Guo et al., 2009; Shao et al., 2010a). More importantly, the high negative potential employed in the electrochemically reduced graphene oxide (ErGO) can efficiently reduce the oxygen-rich functional groups present on the GO surface (Wang et al., 2012). Up to now, various electrochemical methods, including cyclic voltammetry (CV) (Chen et al., 2011) and the potentiostatic method (Kong et al., 2013), have been employed. However, the pulsed electrodeposition method (Davies et al., 2011), having some advantages of simplicity, cost efficiency, time saving, and in the production of high-purity deposits, has rarely been applied in the ErGO field till now.

Pillararenes (Ogoshi et al., 2008, 2016; Cao et al., 2009, 2014; Cragg and Sharma, 2012; Xue et al., 2012; Yao et al., 2012; Si et al., 2014; Cragg, 2018; Wang et al., 2019), as a relatively new class of macrocyclic hosts, have received continuous attention owing to their symmetrical rigid pillar-shaped structures, adjustable cavity size, easy functionalization, and unique host-guest recognition capabilities. Practically, a series of pillararenes with good water solubility and recognition capability have been applied to fabricate graphene hybrids to improve their water stability and dispersity as well as to enhance their supramolecular recognition capability in many applications, including sensors, luminescence, electrocatalysis, and electronics; they have therefore attracted wide research interest (Zhou et al., 2013a,b,c; Zhang et al., 2014; Ye et al., 2015; Zhou et al., 2015; Liu et al., 2016; Mao et al., 2016; Yu et al., 2017, 2018; Zhao et al., 2017; Hou et al., 2019; Sun et al., 2019; Tan et al., 2019b,c; Tan S. et al., 2019). Recently, a water-soluble cationic pillar[6]arene (CP6) with 12–${\text{NH}}_{3}^{+}$ groups on both rims was designed and synthesized by our group (Duan et al., 2019). CP6 contains not only one hydrophobic cavity but also 12 hydrophilic ammonium groups on both rims, which can produce electrostatic interaction with the negatively charged groups present in GO to form GO-CP6 nanocomposites with potential applications in materials science.

**Scheme 1:**
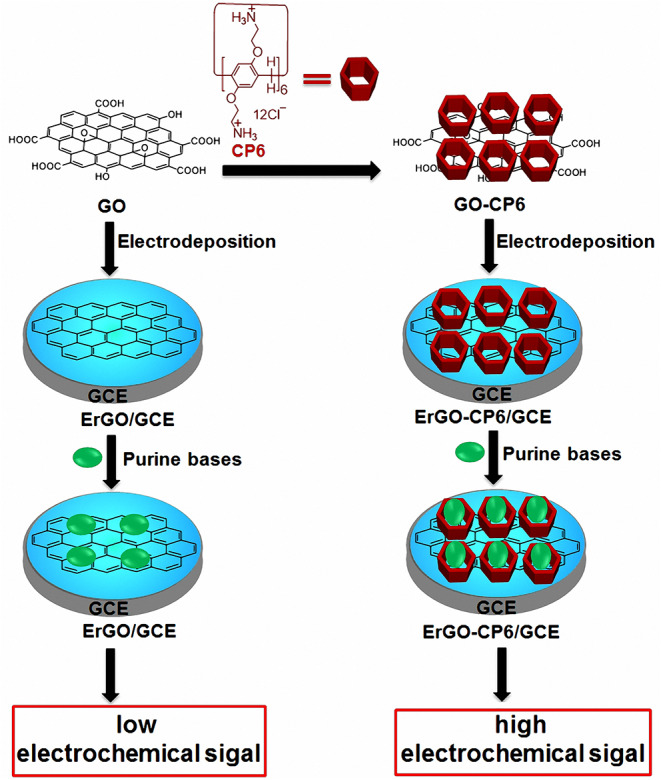
Schematic illustration for the pulsed electrodeposition preparation of ErGO and ErGO-CP6 films on the surface of GCE and sensing the purine bases by an electrochemical strategy.

In this work, we report that CP6 functionalized graphene films were prepared onto glassy carbon electrodes (GCEs) directly from GO-CP6 dispersions by facile one-step pulsed electrodeposition technique (Scheme 1). The electrodeposited nanocomposite films were characterized by scanning electron microscopy (SEM) and Raman spectra. The electrocatalytic activity of the present ErGO-CP6-modified GCE (ErGO-CP6/GCE) was examined by taking five purine bases [adenine (A), guanine (G), xanthine (X), hypoxanthine (HX), and uric acid (UA)] as the probes. The electrochemical behaviors of five purine bases at the ErGO-CP6/GCE displayed higher electrochemical performance than at those of ErGO/GCE and bare GCE, indicating that the CP6-modified graphene films not only show the excellent electrical properties of graphene but also exhibit high inclusion complexation and enrichment capability of CP6 through the formation of host-guest inclusion complexes between CP6 and the five purine bases.

## Materials and Methods

Graphite was obtained from XFNANO Materials Tech Co., Ltd (Nanjing, China). A, G, X, HX, and UA were purchased from Adamas-beta Ltd. All other reagents were analytically pure and were used as supplied without further purification. Doubly distilled water (DDW) was used for preparing all solutions. Fourier transform infrared (FTIR) spectroscopy measurements were conducted on a Thermo Fisher Nicolet 6700. UV-vis spectroscopy data were collected by a Shimadzu UV-3600 spectrophotometer (UV-3600, Shimadzu, Japan). Thermogravimetric analysis (TGA) was performed using NETZSCH STA449F3 thermogravimetric analyzer at a heating rate of 10°C·min^−1^ under nitrogen atmosphere from 30 to 800°C. Raman spectra were recorded using an inVia Reflex Raman spectrometer (Renishaw Co., England). A Quanta-250 scanning electron microscope (SEM) (FEI, Czech) was used for imaging. X-ray photoelectron spectroscopy (XPS) data were collected with Thermo Fisher Scientific ESCALAB-250XI spectrometer. Al K alpha radiation was used as an X-ray source (1486.6 eV). Zeta potential measurements were conducted on a Malvern Zetasizer Nano series. All fluorescence titration experiments were conducted on a Cary Eclipse fluorescence spectrophotometer (Agilent, Australia) at room temperature. All electrochemical experiments were carried out using a CHI 650A electrochemical analyzer (CHI Instrument, China) and RST5000 electrochemical workstation (Zhengzhou Shiruisi Technology, China). A conventional three-electrode system was employed, where a saturated calomel electrode (SCE) served as the reference electrode, a platinum (Pt) wire electrode as the auxiliary electrode, and the modified GCE (d = 3.0 mm) as the working electrode. All pH values were measured with a PHS-3C digital pH meter (Shanghai Leici Instrument Factory, Shanghai, China), which was calibrated daily at 25°C.

### Preparation of GO-CP6 Composite

GO was prepared from natural graphite powder by a modified Hummer's method (Hummers and Offeman, 1958), and CP6 was prepared according to our previously published procedure (Duan et al., 2019). A GO-CP6 composite was prepared: CP6 (6 mg) and GO (6 mg) were dissolved in 10 mL of DDW by sonication for 10 min, and then the mixture reacted for 12 h at room temperature under continuous stirring. The black dispersion was separated by centrifuging at 18,000 rpm for 20 min, thoroughly rinsed with DDW three times, and dried under vacuum to obtain GO-CP6 composite. The GO-CP6 powder, which can be easily dispersed in a 0.2 M pH 6.8 PBS by ultrasonication again was obtained by freeze drying for further characterization.

### Pulsed Electrodeposition Preparation of ErGO and ErGO-CP6 Films Onto GCE

Prior to use, the GCE surface was successively polished with 0.3 and 0.05 μm Al_2_O_3_ powder and washed thoroughly with DDW between each polishing step, and then the polished GCE was sonicated in ethanol and DDW for 2 min prior to each experiment before then being dried under N_2_ blowing. After drying, the cleaned GCE was immersed in the aforementioned PBS (pH 6.8) containing 0.8 mg·mL^−1^ GO-CP6, and the GO-CP6 was electrodeposited onto the GCE by a pulse potentiostatic method under constant stirring at room temperature. The optimum pulse electrodeposition parameters were set: anodic potential, −0.1 V; cathodic potential, −1.3 V; anodic pulse duration time, 0.7 s; cathodic pulse duration time, 0.3 s; and the total experimental time, 100 s. After electrodeposition, the ErGO-CP6/GCE was thoroughly washed with DDW and then kept under ambient conditions prior to use. For comparison purposes, we also prepared ErGO/GCE through the similar pulse potentiostatic method except that the anodic potential of 0.1 V was used to deposit GO on GCE.

## Results and Discussion

### Characterization of GO-CP6 Composite

**Figure 1:**
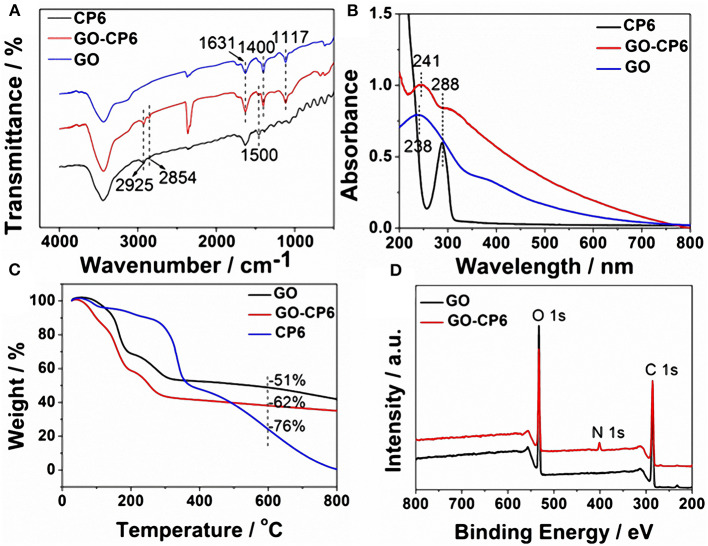
Characterization of materials. FTIR spectra **(A)**, UV-vis absorption spectra **(B)**, and TGA curves of CP6, GO-CP6, and GO **(C)**. XPS survey spectra of GO and GO-CP6 **(D)**.

FTIR spectra supported the successful functionalization of GO with CP6. As can be seen from Figure 1A, the FTIR spectrum of GO displays the stretching vibrations of –OH (3,436 cm^−1^), C=C (1,631 cm^−1^), C-OH (1,400 cm^−1^), and C-O (1,117 cm^−1^). In the spectrum of GO-CP6, the new bands observed at 2,925 and 2,854 cm^−1^ correspond to asymmetric and symmetric CH_2_ stretching vibrations, respectively, and the bands centered at 1,500 cm^−1^ are observed which are assigned to the typical CP6 absorption features of the phenyl stretching vibrations, indicating that the CP6 molecules have been successfully attached to the surface of GO (Allen et al., 2010). The successful preparation of GO-CP6 composites is further confirmed by UV-vis absorption spectra. As shown in Figure 1B, the characteristic absorption peak of CP6 is located at 288 nm. The absorption peak of GO is about 238 nm. When CP6 was loaded onto GO, GO-CP6 composites present two main absorption peaks, which are assigned to the absorption peaks of CP6 (~288 nm) and GO (241 nm). Therefore, the successful chemical modification of CP6 on GO is further confirmed by UV-vis absorption spectra.

TGA measurement was further used to determine the mass fraction of CP6 in GO-CP6 composites. As shown in Figure 1C, pure CP6 slowly decomposed at ~300°C. The GO has a mass loss (51%) at ~600°C because of the pyrolysis of the labile oxygen-containing functional groups. The loss in mass of the GO-CP6 was about 62 wt% at ~600°C. The mass loss caused by CP6 decomposition was evaluated to be 11 wt% by deducting the mass loss of the GO, suggesting that the mass fraction of CP6 molecules loaded on the surface of GO is 11 wt%. This result is exciting because GO loading plentiful CP6 molecules will provide a good opportunity to expand the inclusion complexation and enrichment ability of CP6.

To further illustrate the formation of GO-CP6, XPS analysis was also performed to determine the compositions of GO and GO-CP6. As shown in Figure 1D, a significant N1s peak was observed for the GO-CP6 sample, which comes from the –${\text{NH}}_{3}^{+}$ groups of CP6, but there was no N signal on the GO, further revealing the successful loading of CP6 onto GO.

**Figure 2:**
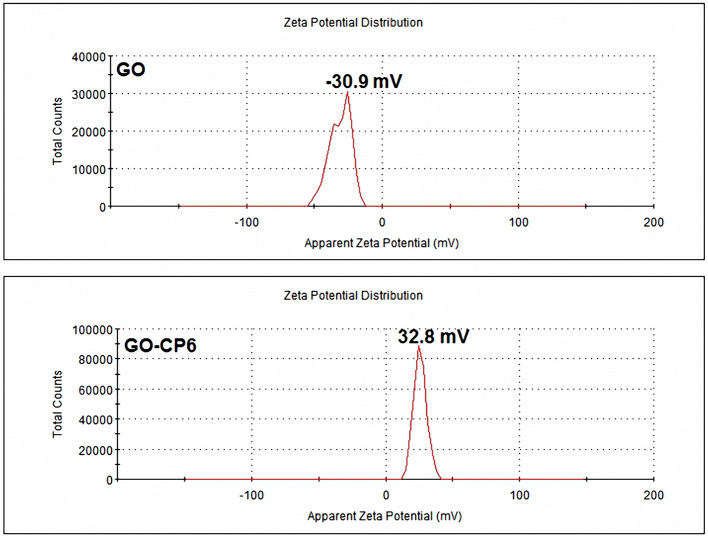
Zeta potentials of GO and GO-CP6.

The average zeta potentials of GO and GO-CP6 are −30.9 and 32.8 mV, respectively, as shown in Figure 2. Compared to the zeta potential of GO, the zeta potential of GO-CP6 increases by ~63.7 mV, and this is caused by the introduced positive charges of –${\text{NH}}_{3}^{+}$ in the CP6 molecule. The introduced positive charges in GO-CP6 facilitate the stability of nanocomposite owing to the increased repulsion of positive charges. Furthermore, the zeta potential of GO-CP6 is higher than 30 mV, suggesting that the stability and dispersion of GO-CP6 are very high (Fu et al., 2013). Therefore, these results of FTIR, UV-vis, TGA, XPS, and zeta potential suggest that CP6 has been successfully grafted on the surface of GO.

### Pulsed Electrodeposition of ErGO and ErGO-CP6 Films on GCE

GO colloids are negatively charged in weak acid solution (Chen et al., 2011), while the surface charge of GO-CP6 is positively charged in weak acid solutions (Figure 2). When positive and negative potentials were applied on the GCE, respectively, GO and GO-CP6 could be spontaneously deposited onto the surface of GCE due to the strong electrostatic attraction. In accordance with the literature (Chen et al., 2011), the as-deposited GO can be electrochemically reduced at *E* = −1.1 V vs. SCE. Herein, pulse potentiostatic method was used to achieve the electrodeposition of ErGO and ErGO-CP6 films in which 0.1 and −0.1 V vs. SCE were used to deposit GO and GO-CP6 on GCE, respectively, followed by employing −1.3 V vs. SCE to electrochemically reduce the as-deposited GO and GO-CP6 to ErGO and ErGO-CP6.

### Characterization of the ErGO/GCE and ErGO-CP6/GCE

**Figure 3:**
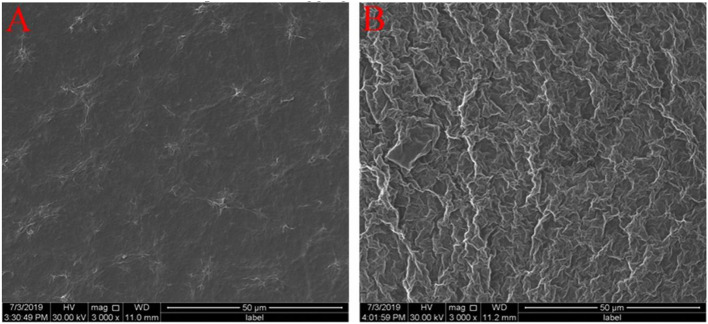
SEM images of ErGO films **(A)** and ErGO-CP6 films **(B)** modified GCE.

The surface morphologies of ErGO and ErGO-CP6 films electrodeposited on GCE were examined by SEM. Figure 3 shows the SEM images of the ErGO and ErGO-CP6 films electrodeposited on the surface of GCE. As can be seen from Figure 3A, the ErGO films exhibit a curly morphology consisting a thin wrinkling paper-like structure and distribute homogeneously on the surface of GCE. In contrast, the ErGO-CP6 films (Figure 3B) possess more crumpled sheets closely associated with each other. Furthermore, aggregation barely occurs on the thin films, suggesting that the electrodeposition of ErGO-CP6 films on GCE by pulse potentiostatic method can obtain well-dispersed ErGO-CP6 films and prevent the aggregation.

**Figure 4:**
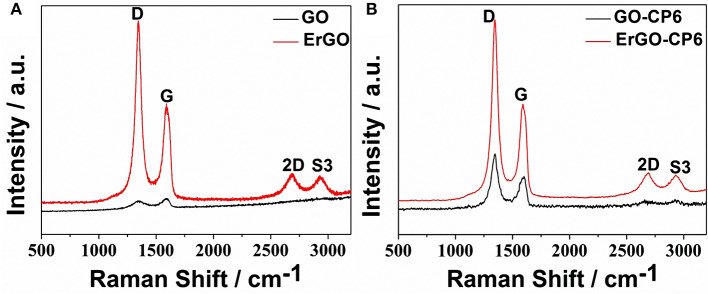
**(A)** Raman spectra of GO and ErGO; **(B)** Raman spectra of GO-CP6 and ErGO-CP6.

Raman spectra of GO, ErGO, GO-CP6, and ErGO-CP6 are shown in Figure 4. The Raman spectrum of GO-CP6 (Figure 4B) displays D and G bands at 1,344 and 1,587 cm^−1^, respectively, which is similar to those of GO prepared through the chemical oxidation of graphite (Figure 4A). The D band at ~1,350 cm^−1^ corresponds to the presence of defects due to sp^3^ hybirdized carbon, while the G band at ~1,575 cm^−1^ indicates the sp^2^ hybirdized carbon (Zhu et al., 2010). The intensity ratio of the D band to the G band (*I*_D_/*I*_G_) of carbon products is generally used to evaluate the extent of disorder or defects that result from vacancies, distortion, and edges (Tuinstra and Koenig, 1970; Stankovich et al., 2007). The larger value of *I*_D_/*I*_G_ is an indication of smaller sp^2^ domains (Tuinstra and Koenig, 1970). After electrochemical reduction of GO and CP6-GO, the Raman spectra both displayed an increase in the intensities of D band compared to those of G band (Figures 4A,B). After the electrodeposition, the *I*_D_/*I*_G_ ratios increased from 0.85 (for GO) to 1.81 (for ErGO) and 1.60 (for GO-CP6) to 1.84 (for ErGO-CP6), respectively, suggesting that smaller sp^2^ carbon domains are formed upon the electrochemical reduction of the GO and GO-CP6 (Stankovich et al., 2007). The two weak and broad 2D bands at ~2,690 cm^−1^ also indicated disorder due to an out-of-plane vibrational mode, and the cooperation between D and G bands also gave rise to an S3 band near 2,932 cm^−1^. The appearance of 2D and S3 bands at ErGO and ErGO-CP6 indicates that electrochemical reduction of GO and GO-CP6 can generate better graphitization compared to chemical reduction (Tung et al., 2009). The above results revealed that the electrochemical reduction of GO and GO-CP6 had indeed taken place, and their electrochemical reduction retained the sp^2^ hybridization of graphene's lattice.

### Electrochemical Characterization of the ErGO/GCE and ErGO-CP6/GCE

**Figure 5:**
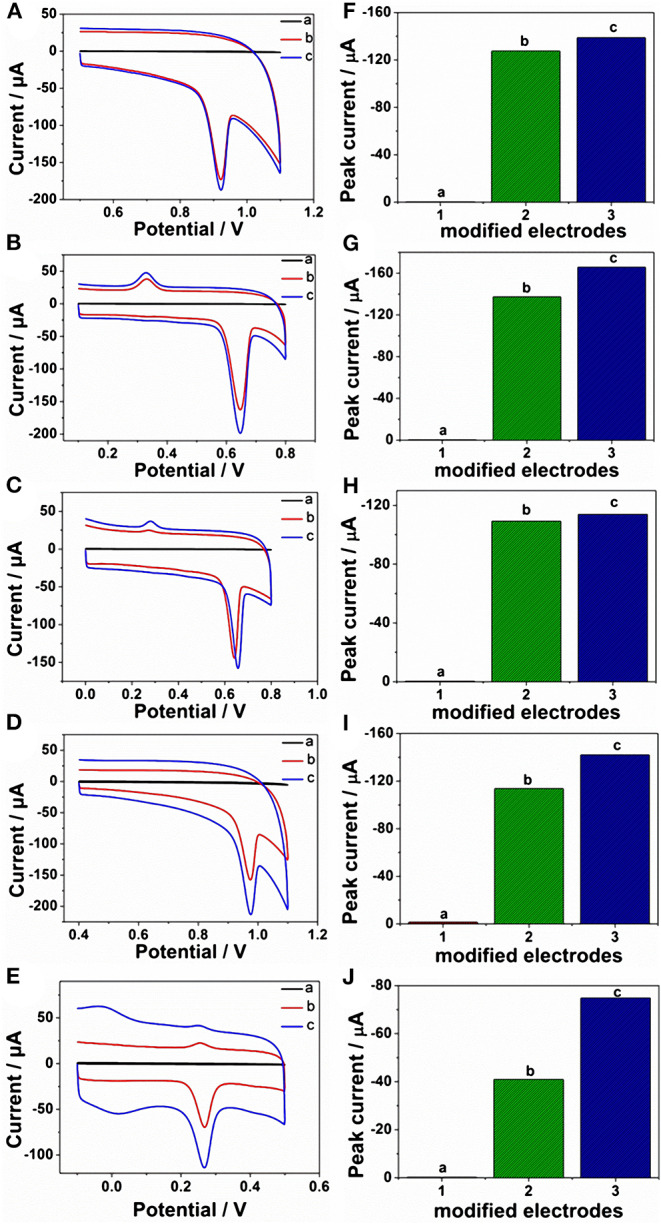
CVs and peak current of CVs of **(A,F)** 5 μM A, **(B,G)** 5 μM G, **(C,H)** 5 μM X, **(D,I)** 5 μM HX, and **(E,J)** 5 μM UA in 0.2 M PBS (pH 6.8) on (a) bare GCE, (b) ErGO/GCE, and (c) ErGO-CP6/GCE. Scan rate: 50 mV·s^−1^.

Given the above discussion, it can be demonstrated that CP6-modified graphene nanocomposite films had been prepared on GCE by pulsed electrodeposition, which could not only improve the stability and dispersion of graphene but also enhance sensitivity for detecting some important biological molecules through supramolecular host-guest complex formation between CP6 and the guest molecules that fit spatially within CP6 cavities. To confirm this conception (Scheme 1), the electrochemical behaviors of five purine bases [adenine (A), guanine (G), xanthine (X), hypoxanthine (HX), and uric acid (UA)] were investigated. CVs and peak currents of the above five purine bases on (a) GCE, (b) ErGO/GCE, and (c) ErGO-CP6/GCE are shown in Figures 5A–J, respectively. As shown in Figures 5A–E (curve a), very weak redox peaks currents on bare GCE were observed for five purine bases. Meanwhile, there were increases in the oxidation peak currents of five purine bases at ErGO/GCE compared to the currents at the bare GCE (Figures 5A–E, curve b), which may be ascribed to excellent conductivity and large surface area of ErGO arising from its specific structure. Much to our excitement, at the ErGO-CP6/GCE (Figures 5A–E, curve c), all the oxidation peak currents were noticeably increased and were ~1.2–1.9 times as much as those on ErGO/GCE. This illustrated that CP6 molecules immobilized on the surface of ErGO with excellent supramolecular enrichment ability can form host-guest complexes with all the examined purine bases (the association constants; see Supplementary Figures 1–4 and Supplementary Table 1). The host-guest interactions between the CP6 and the purine bases can further improve the accumulation effect of ErGO-CP6/GCE and therefore increase the concentration of purine bases on the surface of the modified electrode, which would result in the noticeable enhancement in the oxidation peak current as compared with ErGO/GCE. These phenomena demonstrated that ErGO-CP6/GCE not only displayed the excellent properties of ErGO but also exhibited the outstanding supramolecular inclusion complexation and enrichment capability of CP6. Thus, the enhanced electrochemical reactivity of the above five purine bases at the ErGO-CP6/GCE relative to the reactivity of those at the other two electrodes made the ErGO-CP6/GCE a better choice for the electrochemical detection of the above five purine bases at physiological pH.

**Figure 6:**
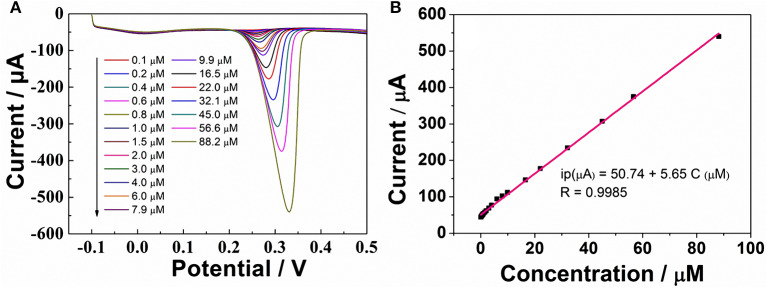
**(A)** DPV response for the different concentrations of UA on ErGO-CP6/GCE in 0.2 M PBS (pH 6.8). **(B)** The calibration curve of UA.

According to the above discussion, ErGO-CP6 is an excellent electrode material for improving the electrochemical response for different purine bases. To evaluate the sensing performance of ErGO-CP6 toward certain purine bases, UA (UA is a vital product of purine metabolism and a crucial biomolecule in biological fluids, such as serum and urine; its detection is of great importance for pathological and physiological diagnosis) was chosen as a representative analyte. Figure 6A displays the differential pulse voltammetric (DPV) response of ErGO-CP6/GCE for different concentration additions of UA. Under the optimized conditions, the DPV response of UA is linearly proportional to the concentration within 0.1–88.2 μM, and the corresponding linear regression equation can be expressed as *I*_pa_ (μA) = 50.74 + 5.65 C_UA_ (μM) with the correlation coefficient (*R*^2^) of 0.9985. The detection limit was estimated as 0.02 μM based on S/N = 3 (Figure 6B). Additionally, we compared our developed sensor with previous reports. As shown in Table 1, the developed ErGO-CP6/GCE electrode is better than or comparable with previously reported UA sensors regarding its analytical parameters, including the detection limit and linear range. The comparative results clearly reveal that the ErGO-CP6/GCE exhibited an excellent electrochemical performance toward the target molecules. Furthermore, the present method of preparation of ErGO-CP6/GCE is easy, simple, and saves time when compared to other carbon-based nanomaterials modified electrode. Therefore, based on a pulsed electrodeposition technique, ErGO-CP6/GCE can be prepared and used as a promising electrode material for sensitive detection of a wide variety of electroactive compounds.

**Table 1:** Comparison with other modified electrodes in the literatures for the detection of UA.

**Modified** **electrode**	**Linear range** **(μM)**	**Detection limit** **(μM)**	**Analytical** **methods**	**References**
Au/RGO^a^/GCE	8.8–530	1.8	DPV	Wang et al., 2014
MoS_2_/PEDOT^b^/GCE	2–25	0.95	DPV	Li et al., 2016
RGO-ZnO^c^/GCE	1–70	0.33	DPV	Zhang et al., 2016
Au-Cu_2_O/RGO/GCE	100–900	6.5	DPV	Aparna et al., 2018
PANI-GO^d^/GCE	2–18	0.2	DPV	Manivel et al., 2013
CNCo^e^/GCE	2–110	0.83	DPV	Liu et al., 2019
AuNPs^f^/GCE	2.8–57.5	2.8	DPV	Shi et al., 2017
ZnO-Au HCs^g^/GCE	10–400	2.375	DPV	Hou et al., 2016
ErGO-CP6/GCE	0.1–88.2	0.02	DPV	This work

a *RGO: reduced grapheme oxide*.

b *PEDOT: poly(3,4-ethylenedioxythiophene)*.

c *RGO-ZnO: reduced graphene oxide-zinc oxide composite*.

d *PANI-GO: polyaniline/graphene oxide*.

e *CNCo: N, Co-doped porous carbon*.

f *AuNPs: Au nanoparticles*.

g *ZnO-Au HCs: ZnO nanorods-Au nanoparticles hybrids composite*.

**Figure 7:**
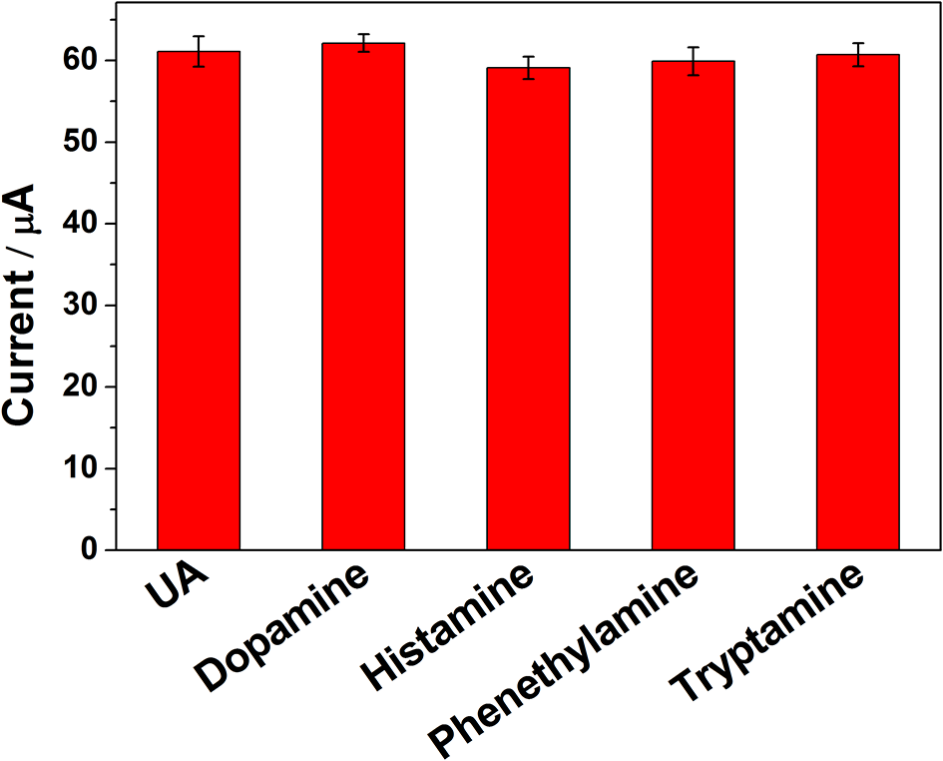
The *i*_pa_ response of ErGO-CP6/GCE in solution containing 2 μM UA in the absence and presence of 10-fold dopamine, histamine, phenethylamine, and tryptamine, using DPV and keeping all the parameters constant.

To evaluate the influence of potential interfering biomolecules, some cyclic biogenic amines, such as dopamine, histamine, phenethylamine, and tryptamine, were studied as a potential interfering compound. Interfering experiments were carried out with 2 μM UA in the absence and presence of 10-fold of dopamine, histamine, phenethylamine, and tryptamine. The observed results (Figure 7) clearly indicated that the peak current of UA was not affected even in the presence of excess concentration of the interfering bioorganic amines, which clearly confirmed that the ErGO-CP6/GCE displayed reasonable selectivity.

## Conclusions

In conclusion, the present work demonstrated a simple, rapid, and green pulsed electrodeposition method for the preparation of ErGO-CP6 films on the surface of GCE. More significantly, due to the unique properties of graphene and CP6, the ErGO-CP6 films at the modified electrode could exhibit outstanding enrichment capabilities and higher electrochemical responses toward A, G, X, HX, and UA than those of ErGO/GCE and bare electrodes. Under optimal conditions, the detection limit of UA was 0.02 μM. The results indicate that the directly electrodeposited reduced graphene oxide-cationic pillar[6]arene composite films may be an attractive and a promising platform for analytical sensing.

## Data Availability Statement

All datasets generated for this study are included in the article/Supplementary Material.

## Author Contributions

QD and KL designed the work. LW and QD made contributions to the experiments and collective data. The paper was written by QD. All authors extensively discussed the results, reviewed the manuscript, and approved the final version of the manuscript to be submitted.

## Conflict of Interest

The authors declare that the research was conducted in the absence of any commercial or financial relationships that could be construed as a potential conflict of interest.
