# Supplementary material for: Facile One-Step Electrodeposition Preparation of Cationic Pillar[6]arene-Modified Graphene Films on Glassy Carbon Electrodes for Enhanced Electrochemical Performance
Source: Front Chem. 2020 Jun 4;8:430. doi: 10.3389/fchem.2020.00430 (PMC7287394; doi:10.3389/fchem.2020.00430)
Supplement: Supplementary file 1 [file Data_Sheet_1.docx]

Supplementary Material

*1. Determination of the association constants between substrates (****A****,* ***G****,* ***X****,* ***HX, UA****) and* ***CP6***

*1.1 Job plot for* ***CP6****⊃* ***A****/****G****/****X****/****HX/UA***


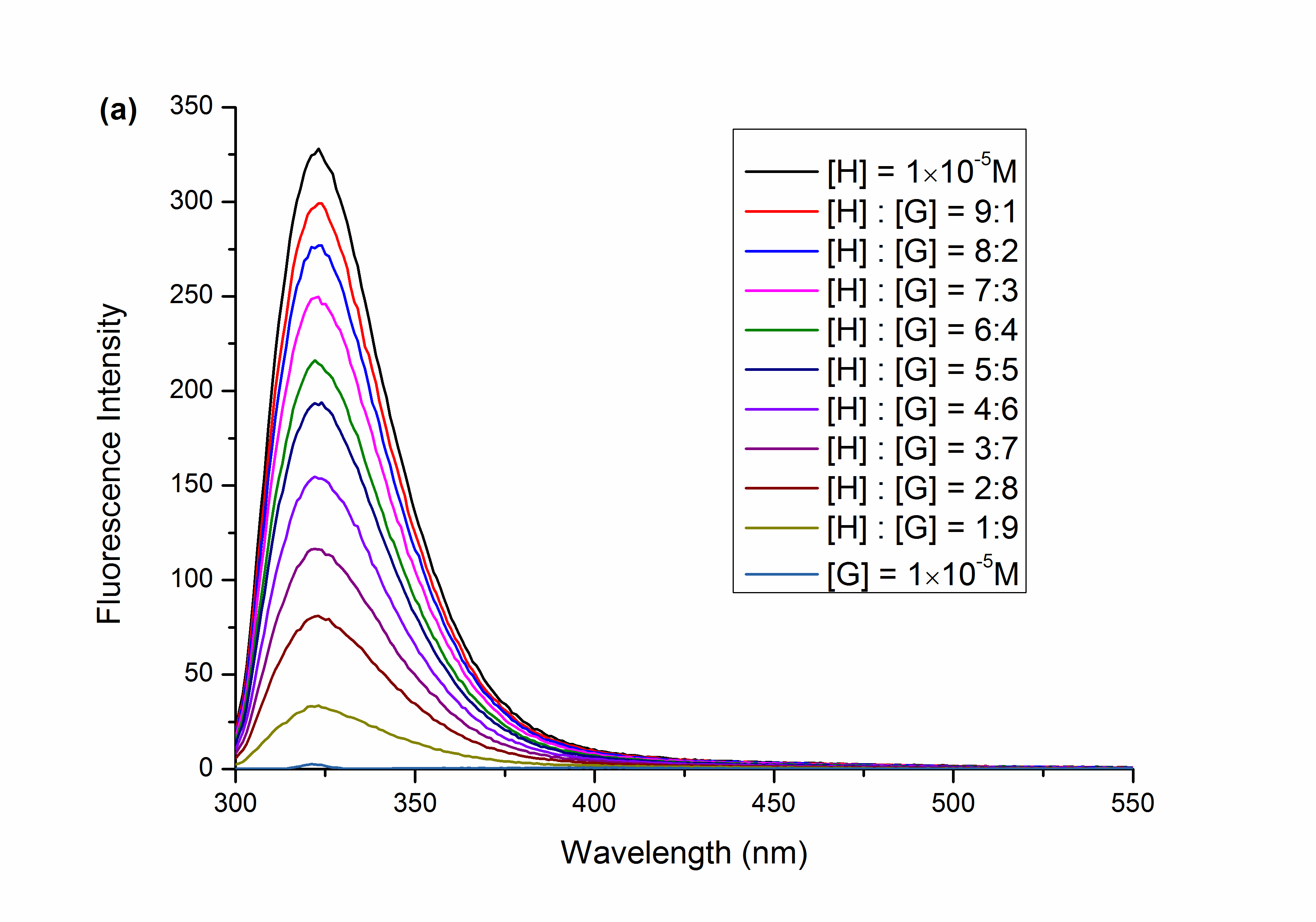

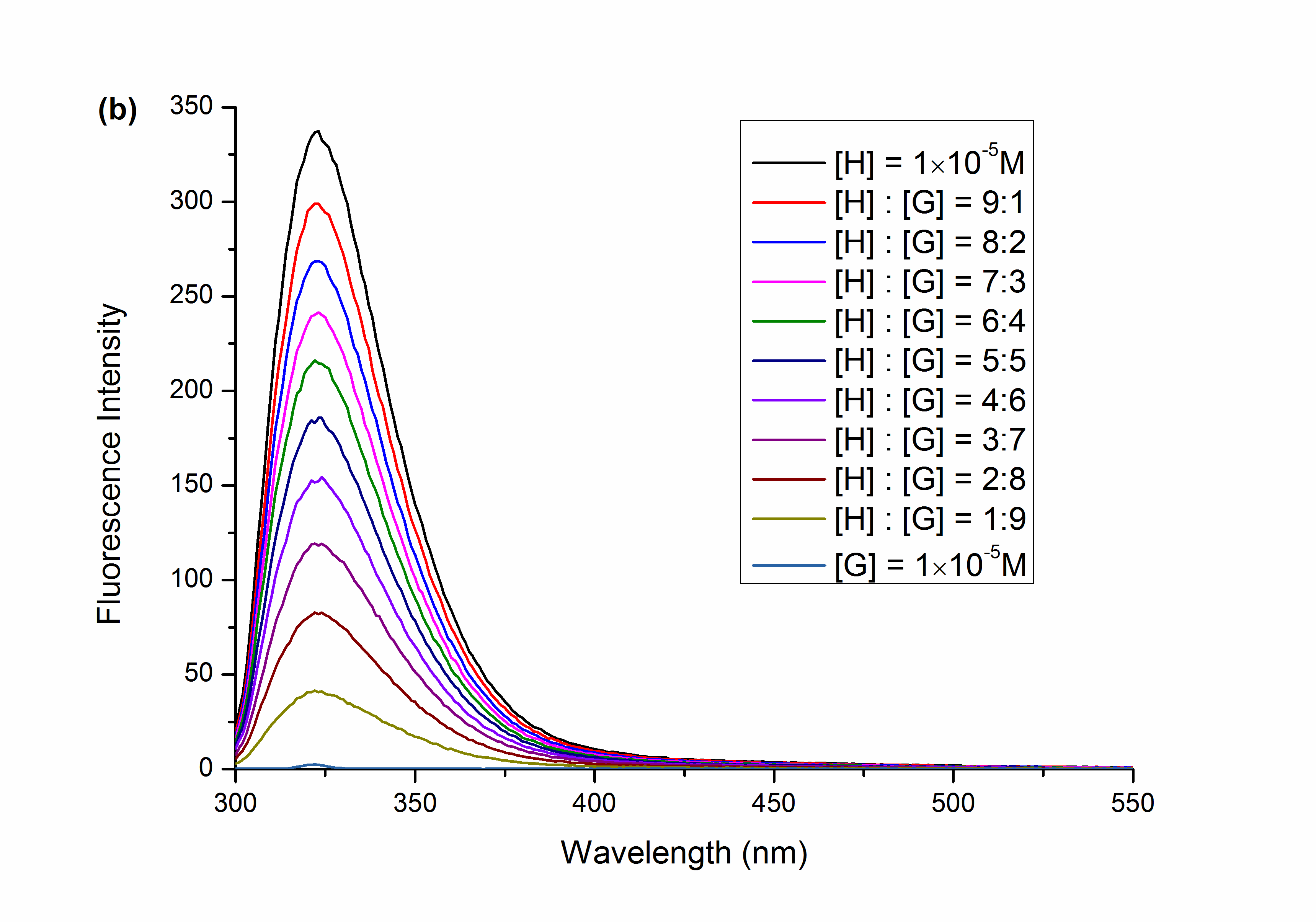


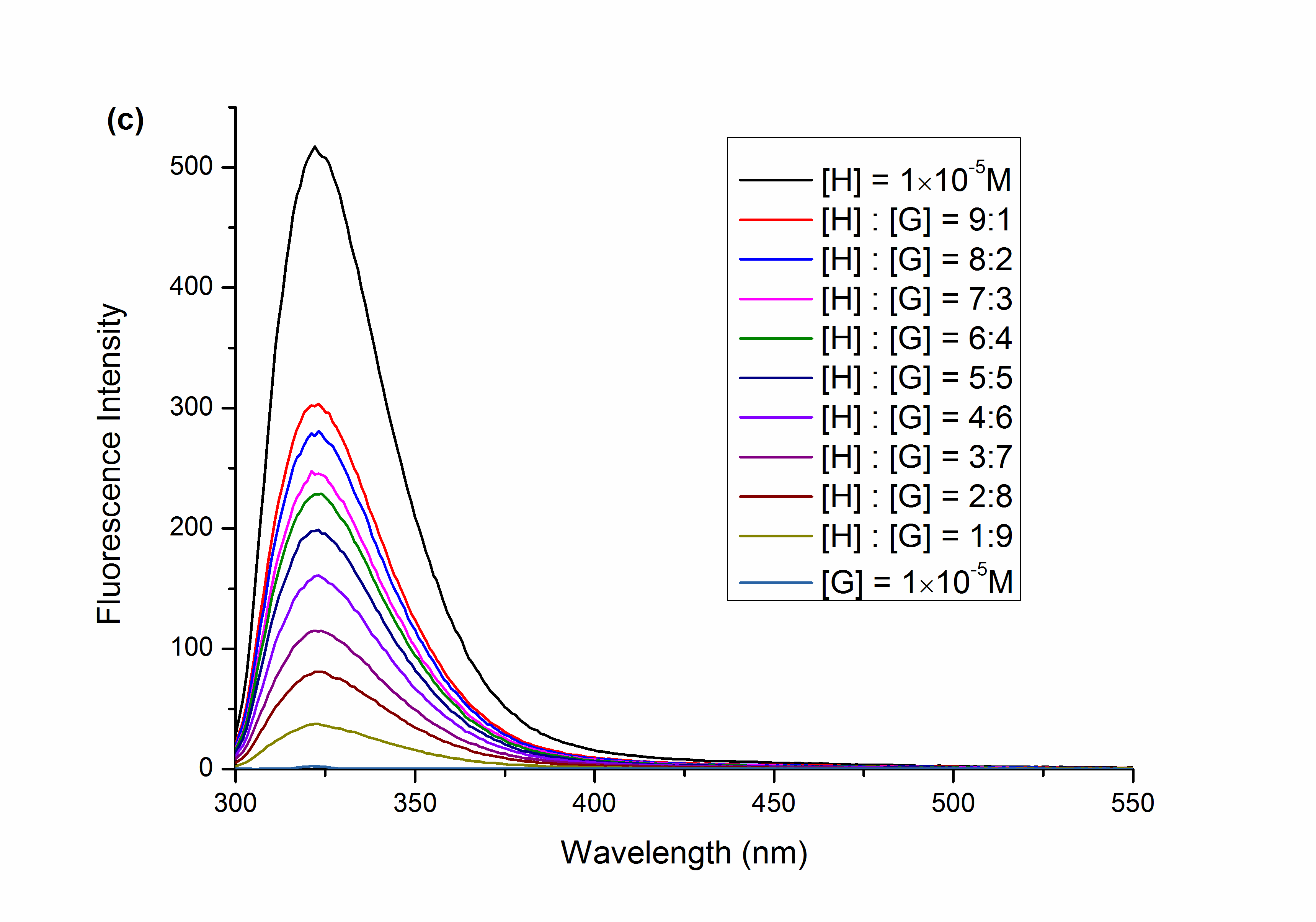

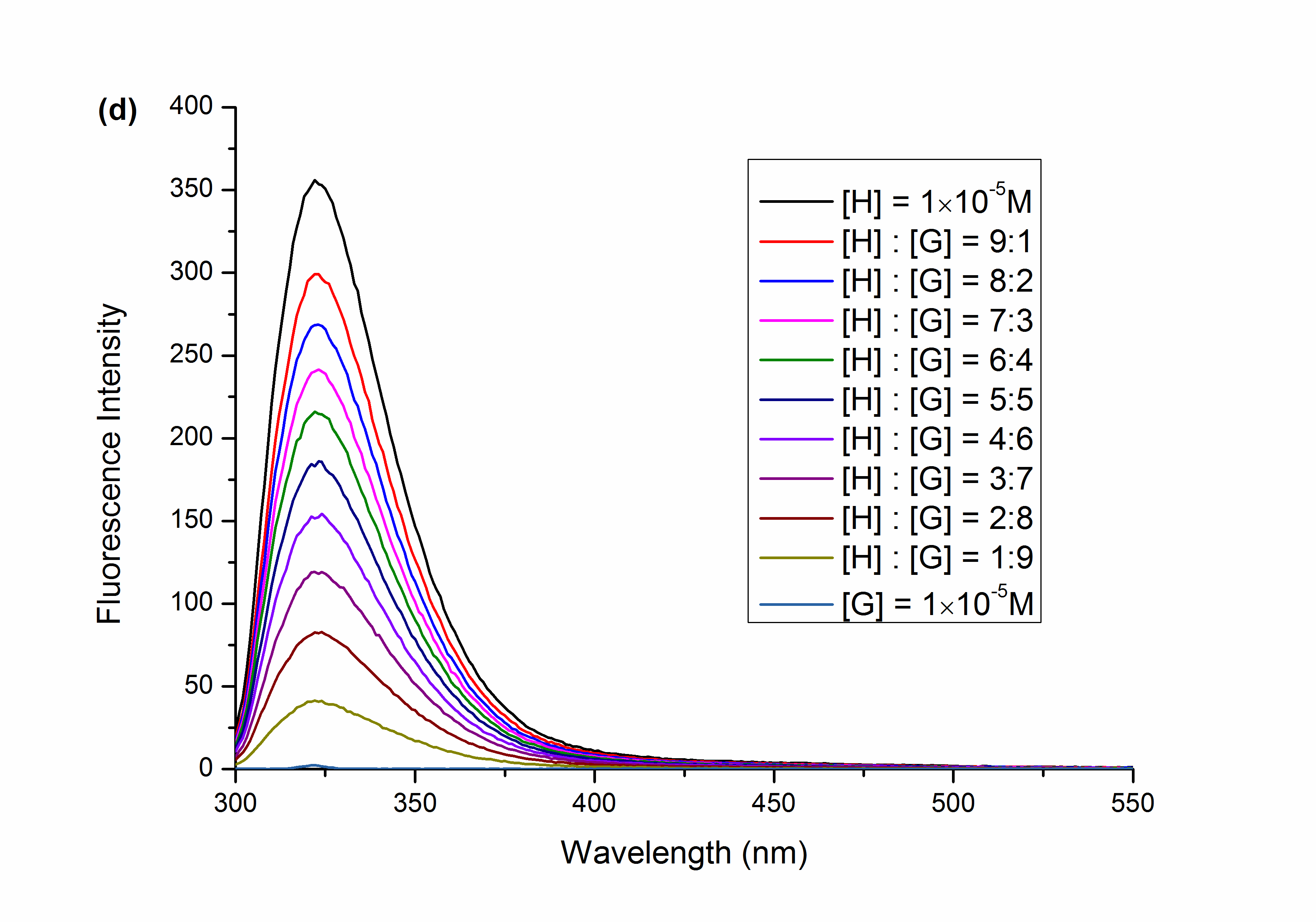


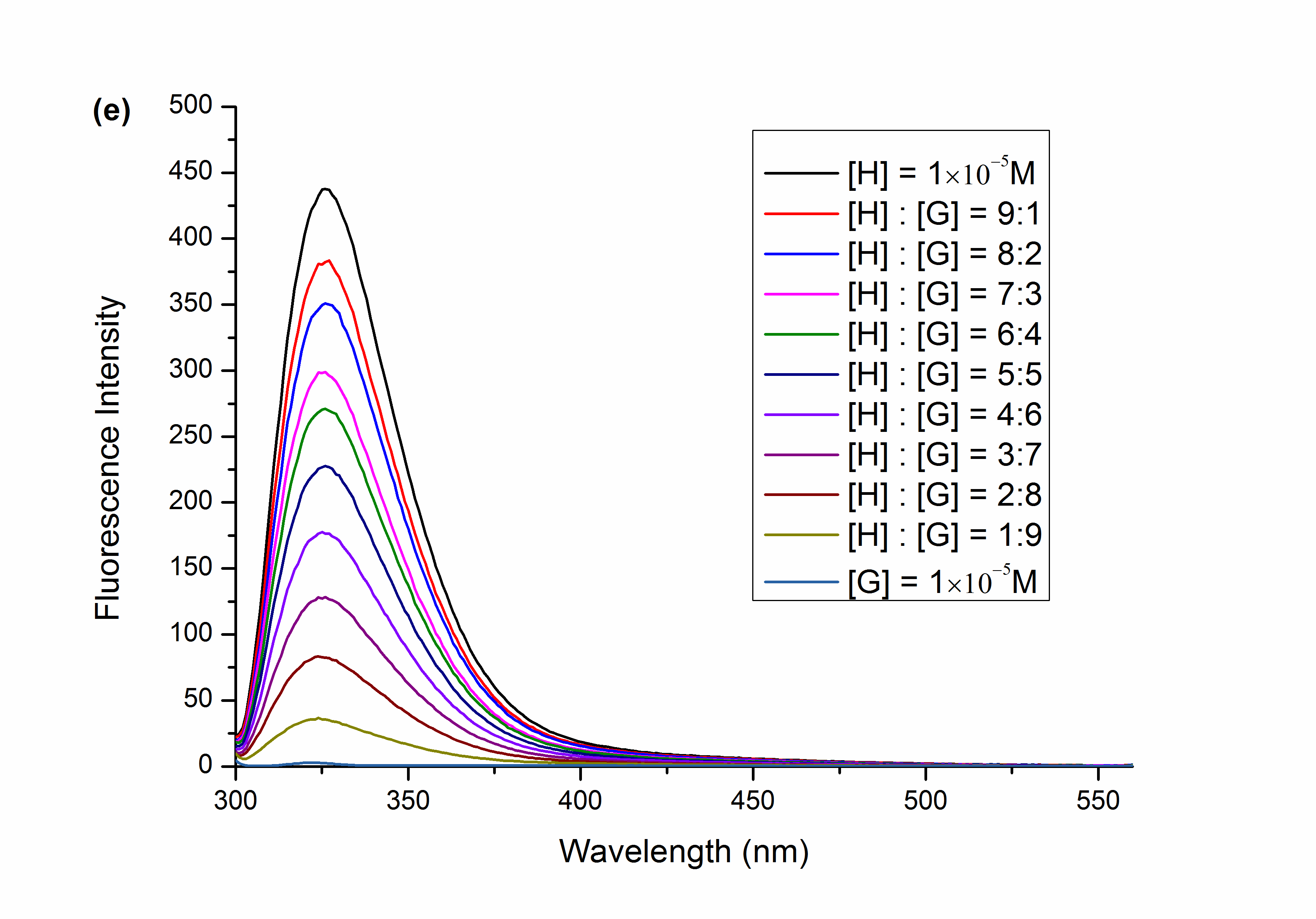


**Supplementary Figure 1.** (a-e) Fluorescence spectra of the mixture of **CP6** and substrates (**A**, **G**, **X**, **HX**, **UA**) in water at different molar ratios while [**CP6**] + [substrates] = 1.0 × 10^−5^ M, respectively.


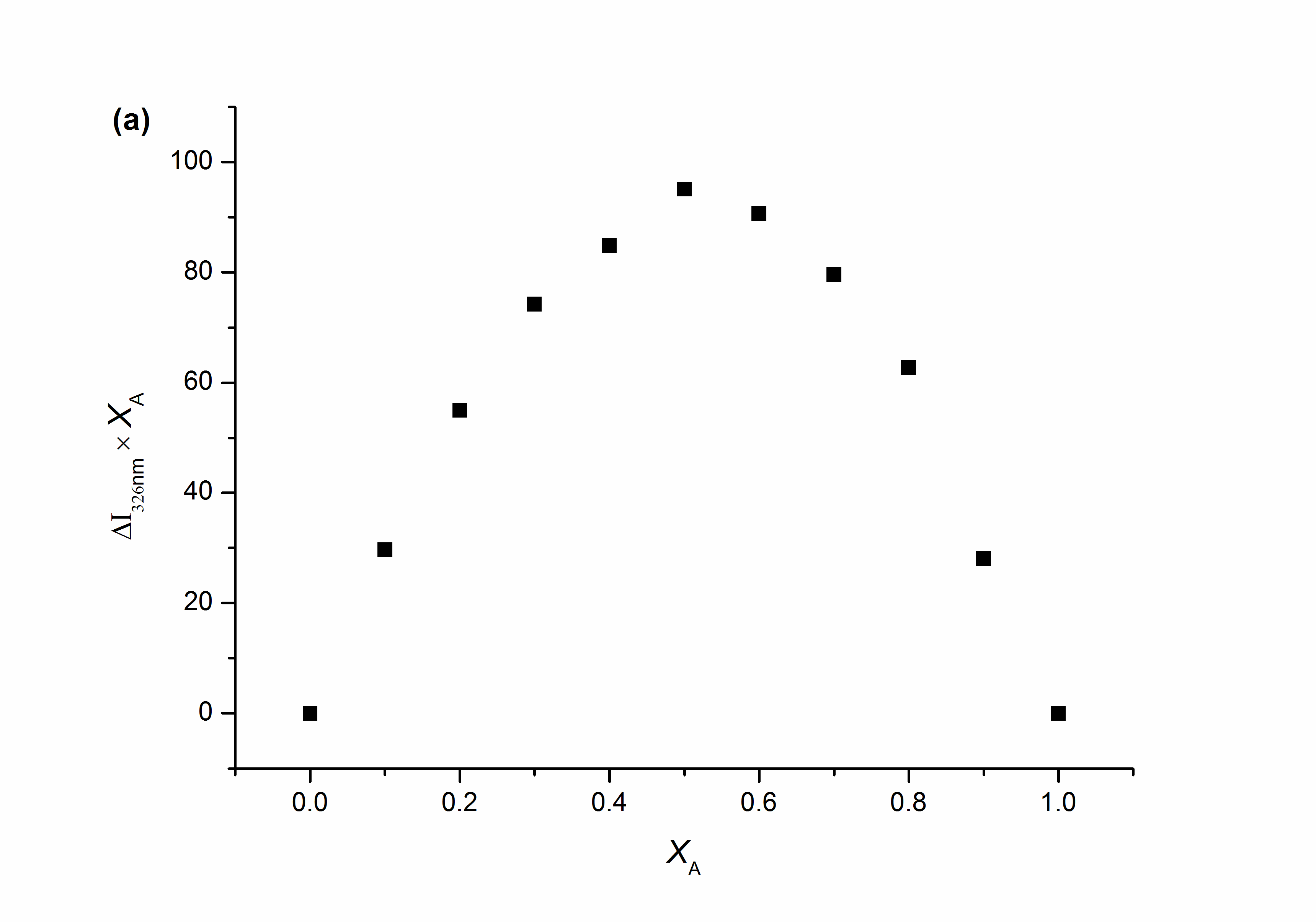

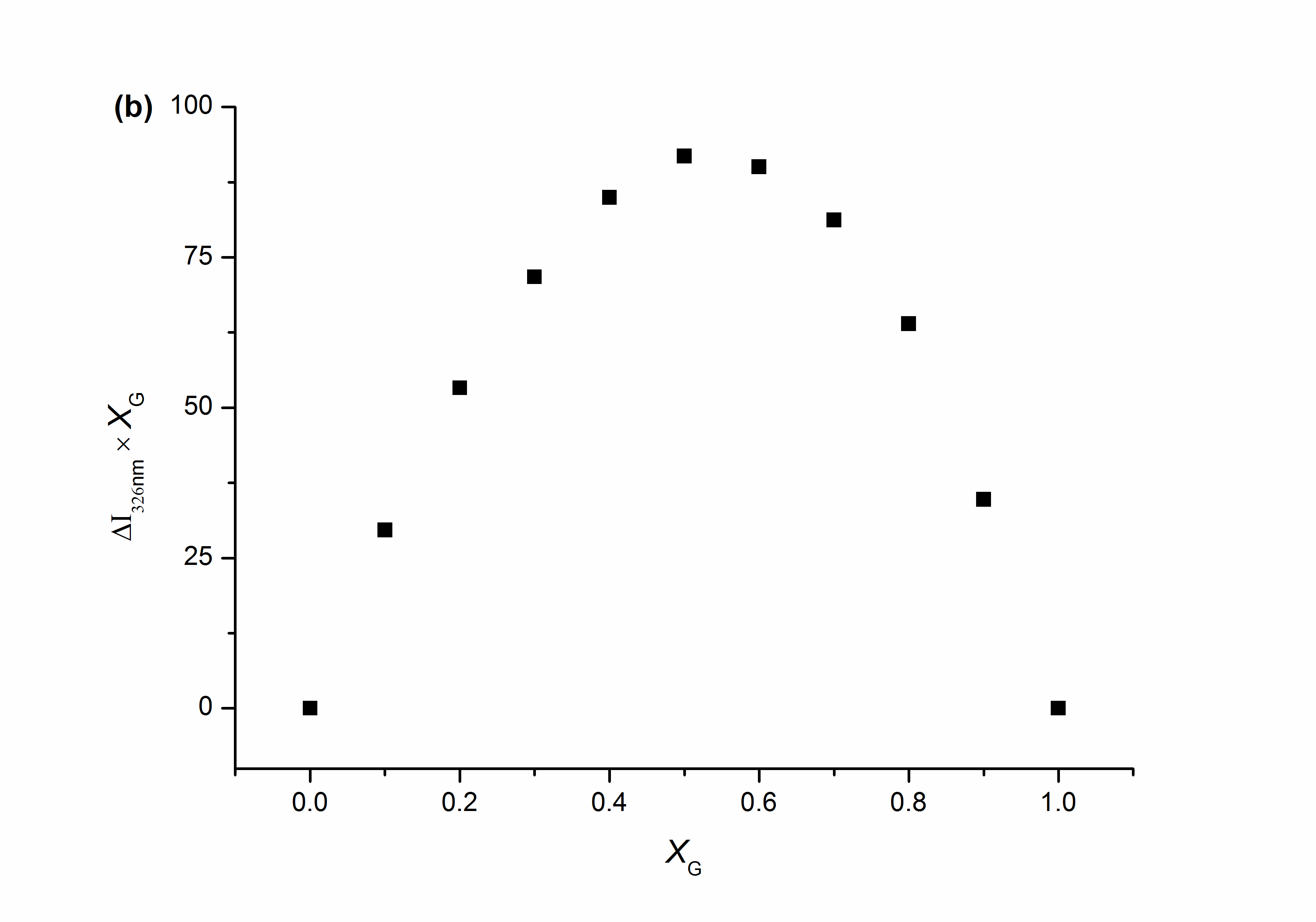


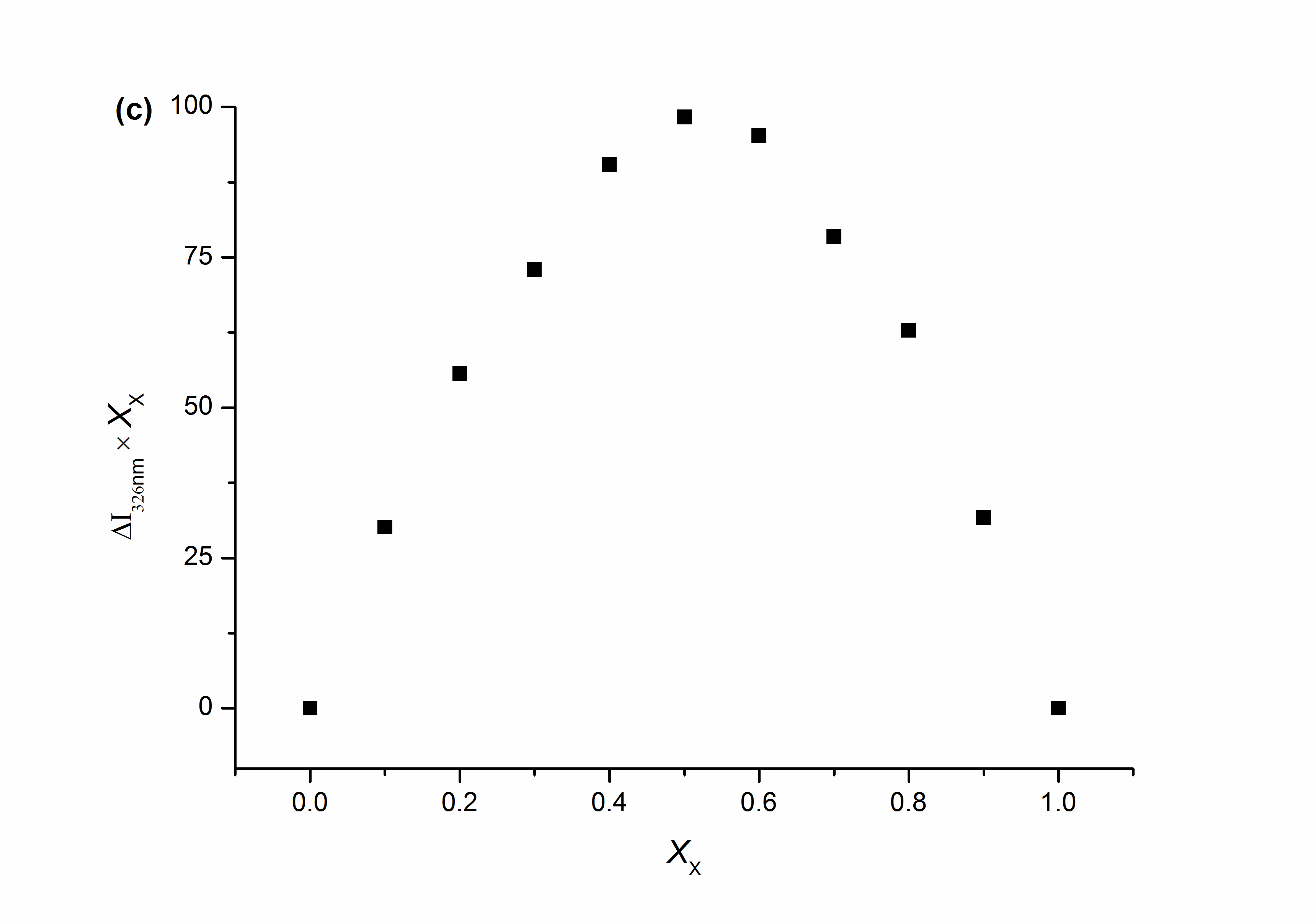

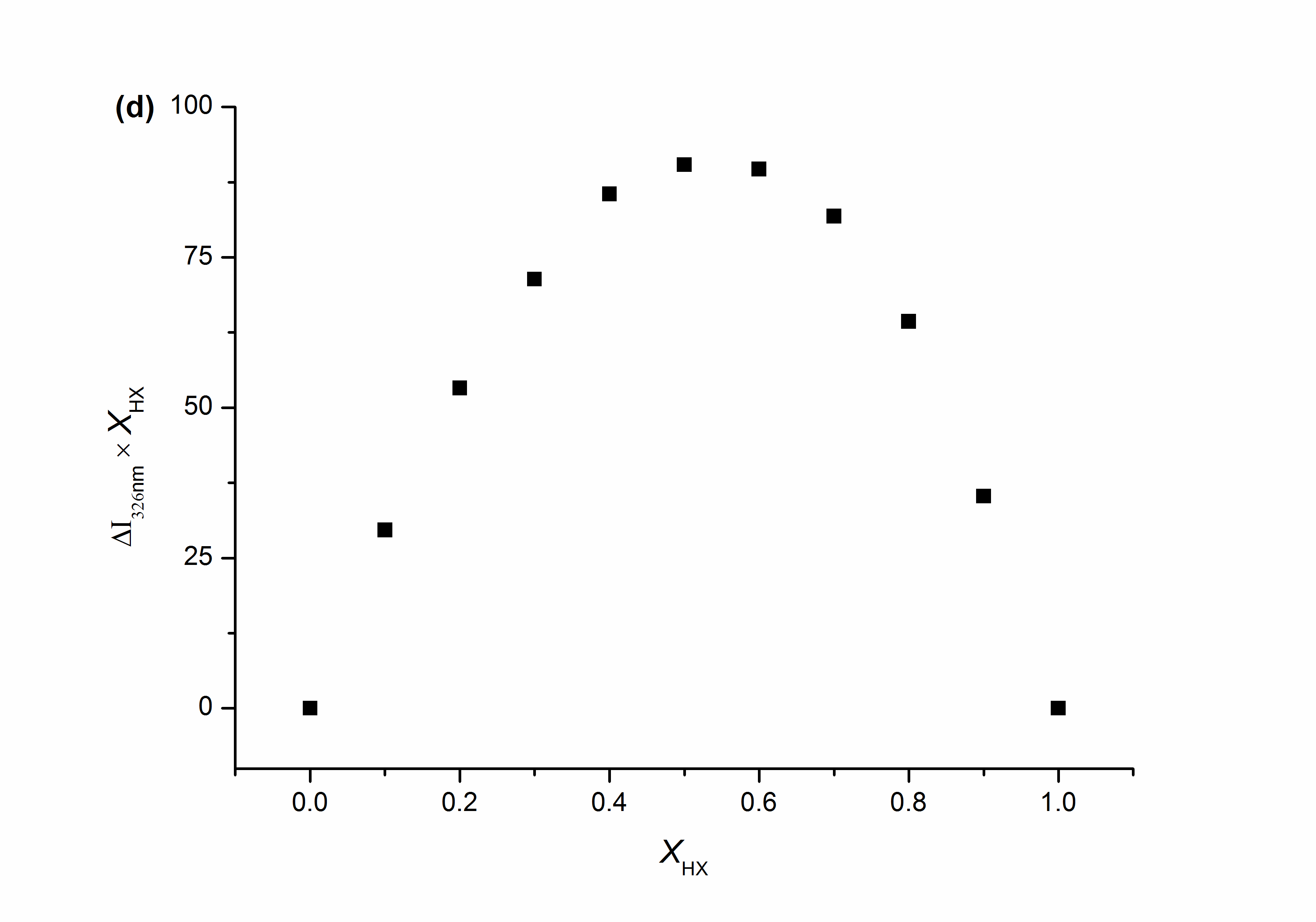


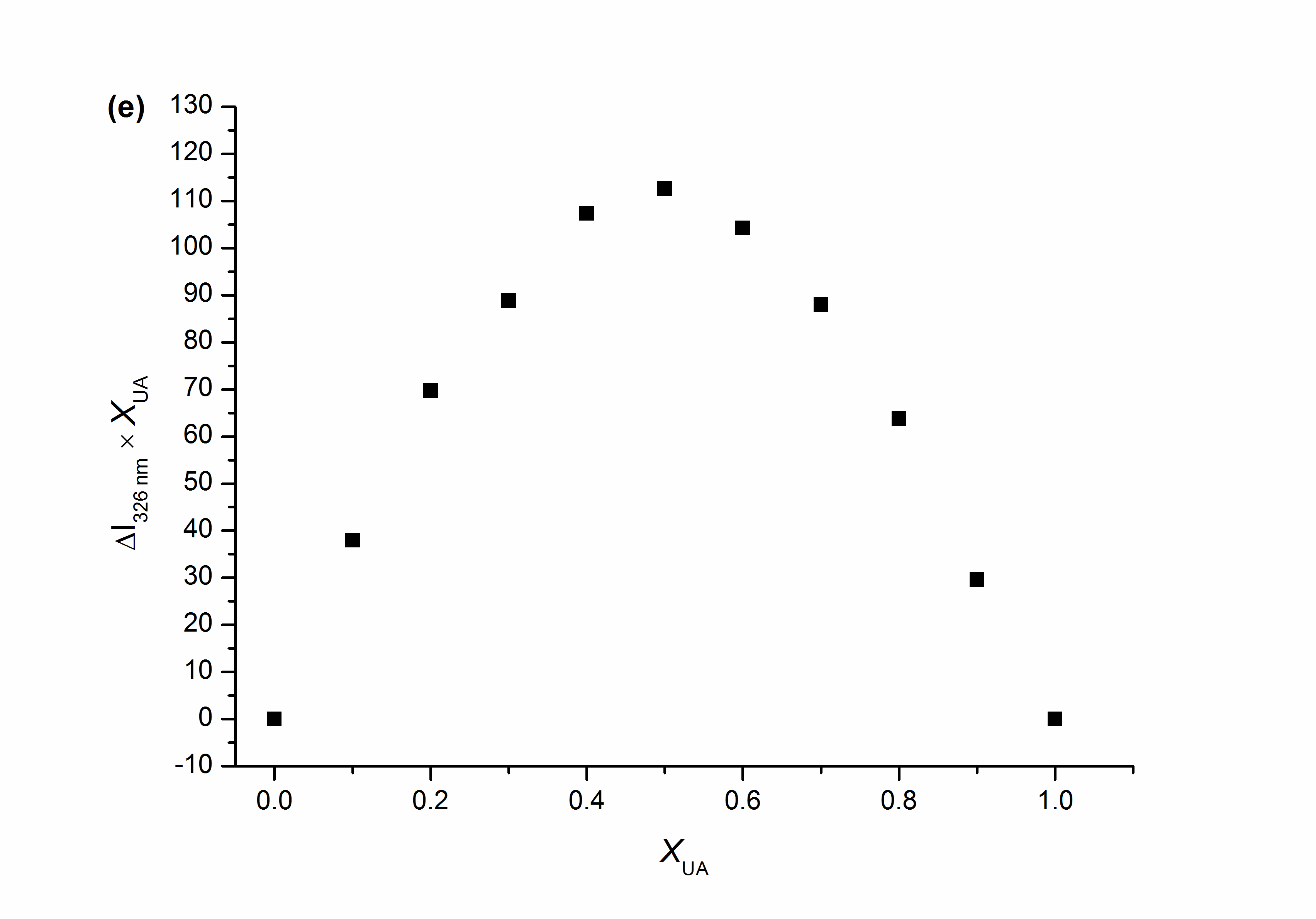


**Supplementary Figure 2.** Job plot showing the 1:1 stoichiometry of the complex between **CP6** and substrates (**A**, **G**, **X**, **HX, UA**) by plotting the difference in fluorescent emission intensity at *λ*_emission_ = 326 nm (*λ*_excitation_ = 290 nm) against the mole fraction of substrates at an invariant total concentration of 0.01 mM in aqueous solution, respectively.

*1.2 Association constants of* ***CP6****⊃* ***A/G/X/HX/UA***

To determine the association constants for the complexation between **CP6** and substrates (**A, G, X, HX,** and **UA**), fluorescence titration experiments were carried out in solutions which had a constant concentration of **CP6** (1.0 × 10^–5^ M) and varying concentrations of substrates. By a non-linear curve-fitting method, the association constants (*K*_a_) of **CP6**⊃**A/G/X/HX/UA** were estimated.

The non-linear curve-fittings were based on the equation:

*ΔF* = (*ΔF*_∞_/[H]_0_) (0.5[G]_0_ + 0.5([H]_0_+1/*K*_a_)−(0.5 ([G]_0_^2^+(2[G]_0_(1/*K*_a_ − [H]_0_)) + (1/*K*_a_ + [H]_0_)^2^) ^0.5^)) (eq. 1)

Where *ΔF* is the fluorescence intensity changes at 326 nm at [H]_0_, *ΔF_∞_* is the fluorescence intensity changes at 326 nm when **CP6** is completely complexed, [G]_0_ is the initial concentration of substrates (**A, G, X, HX, and UA**), and [H]_0_ is the fixed initial concentration of **CP6**.^S1^


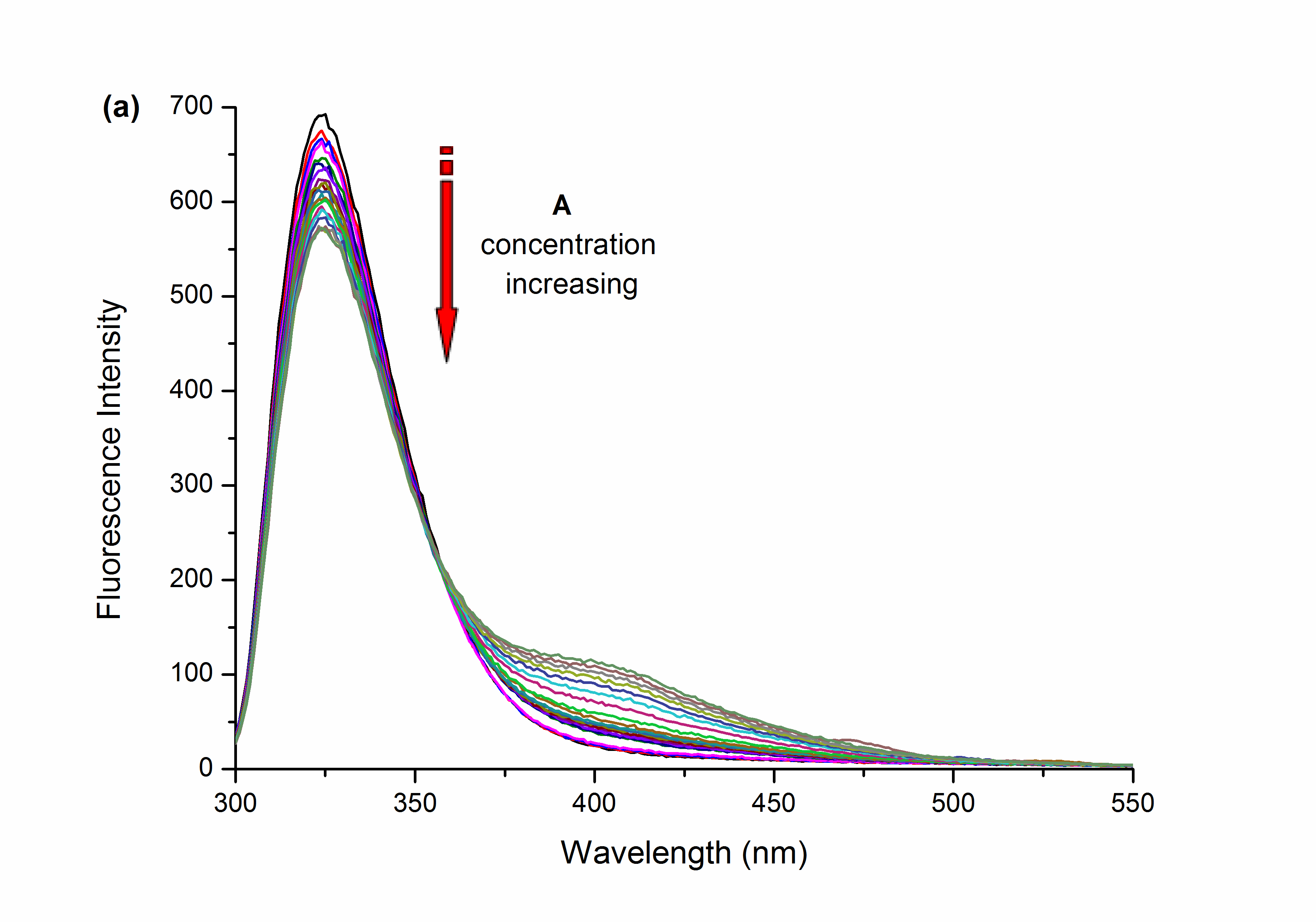

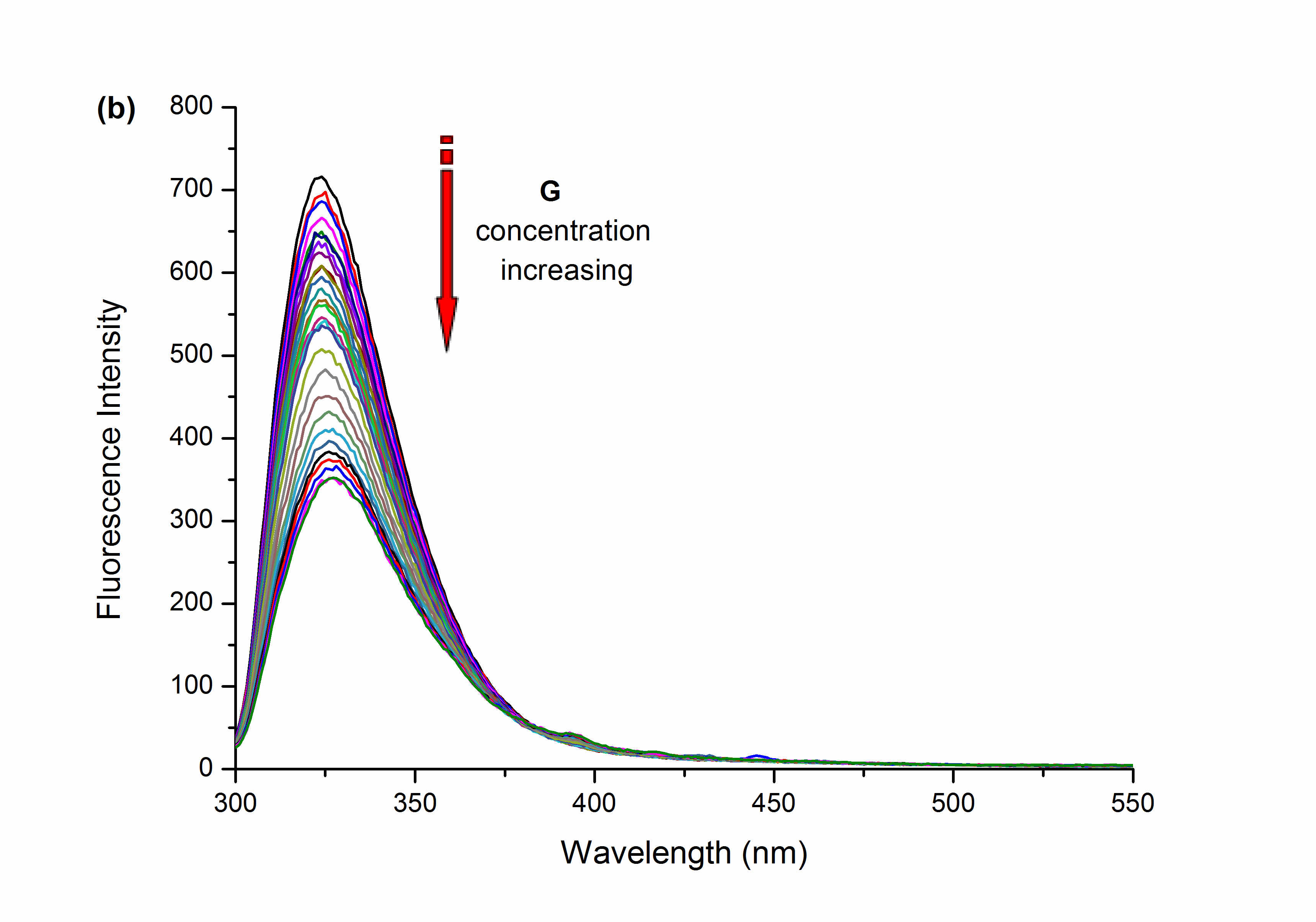


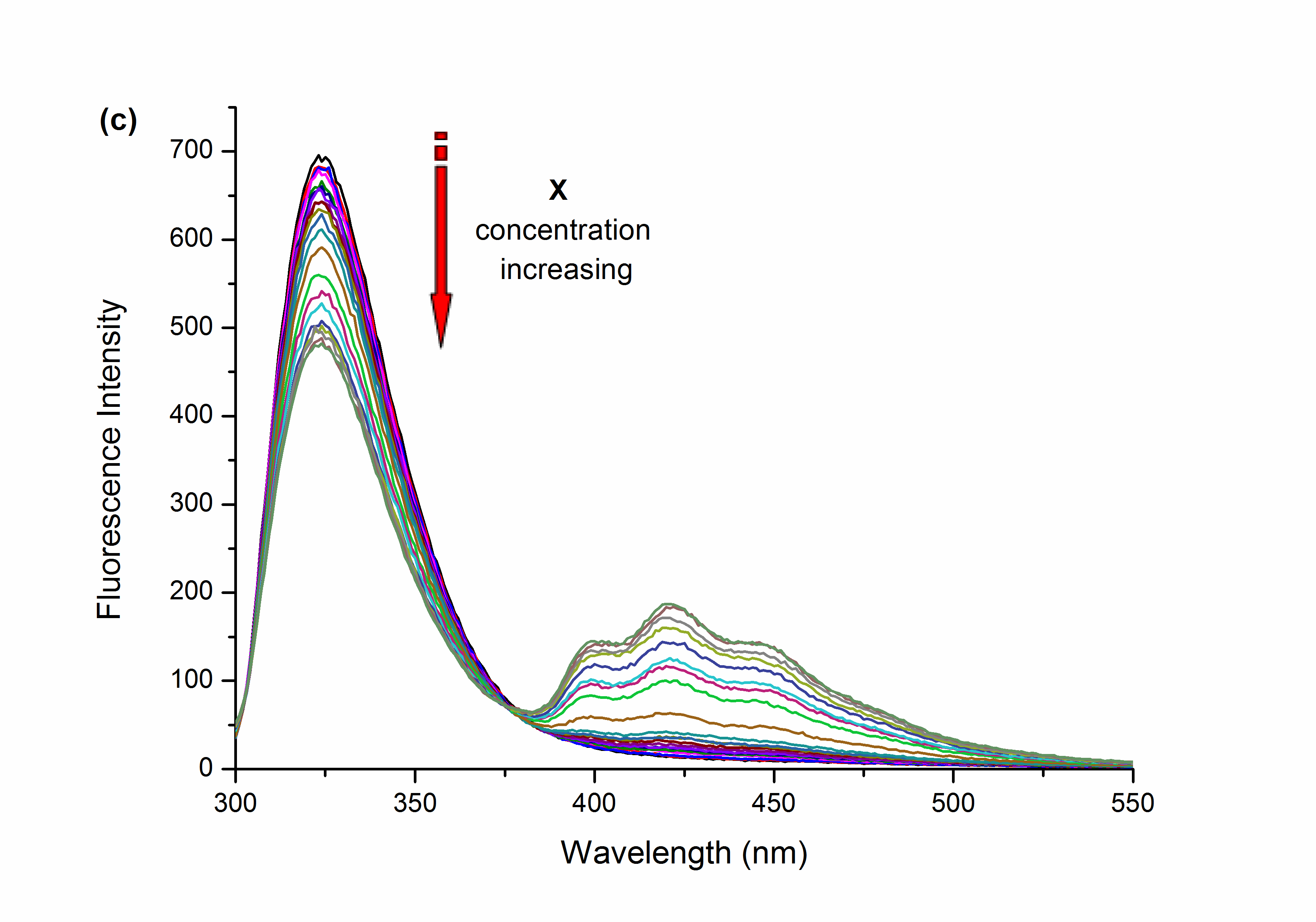

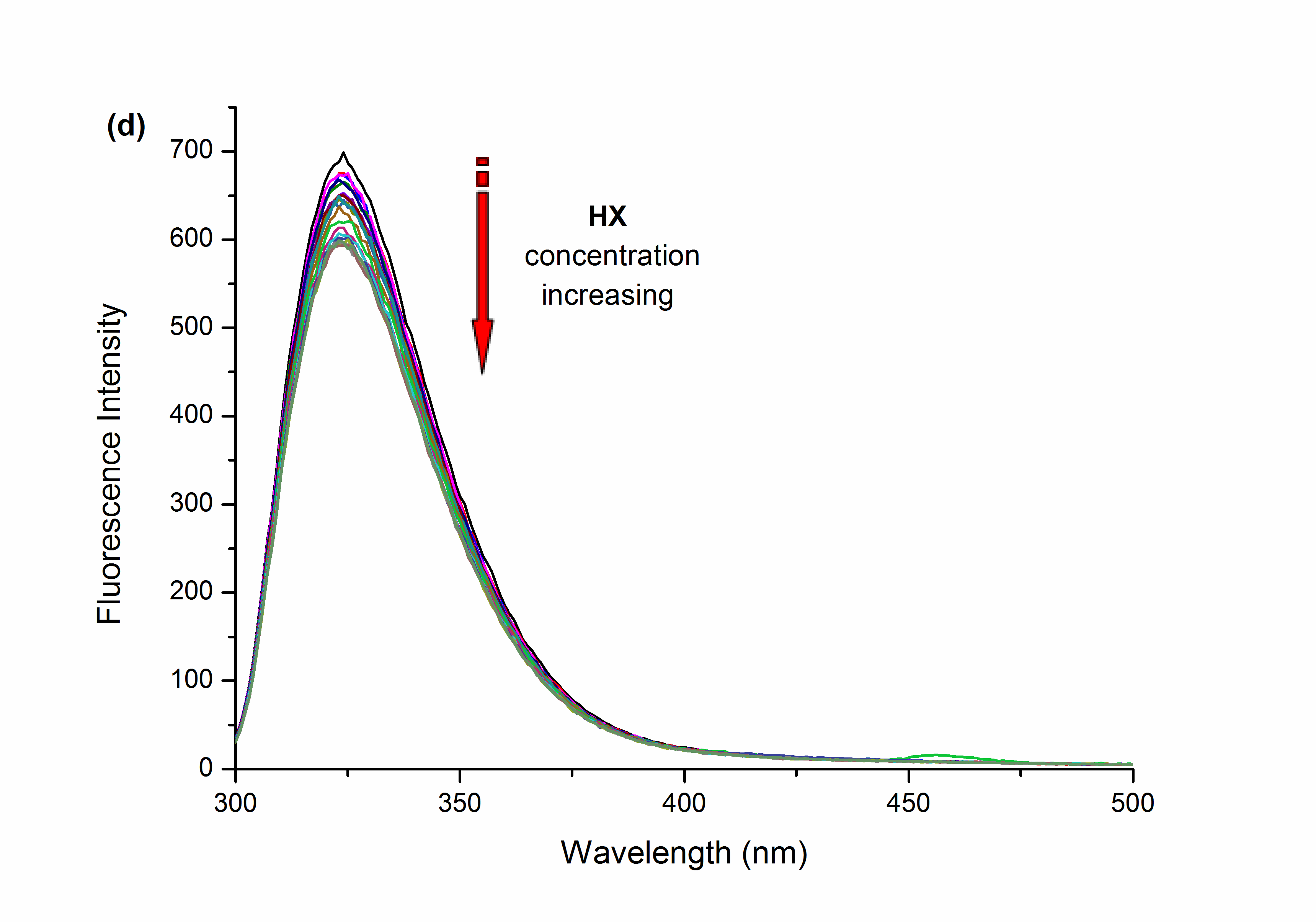


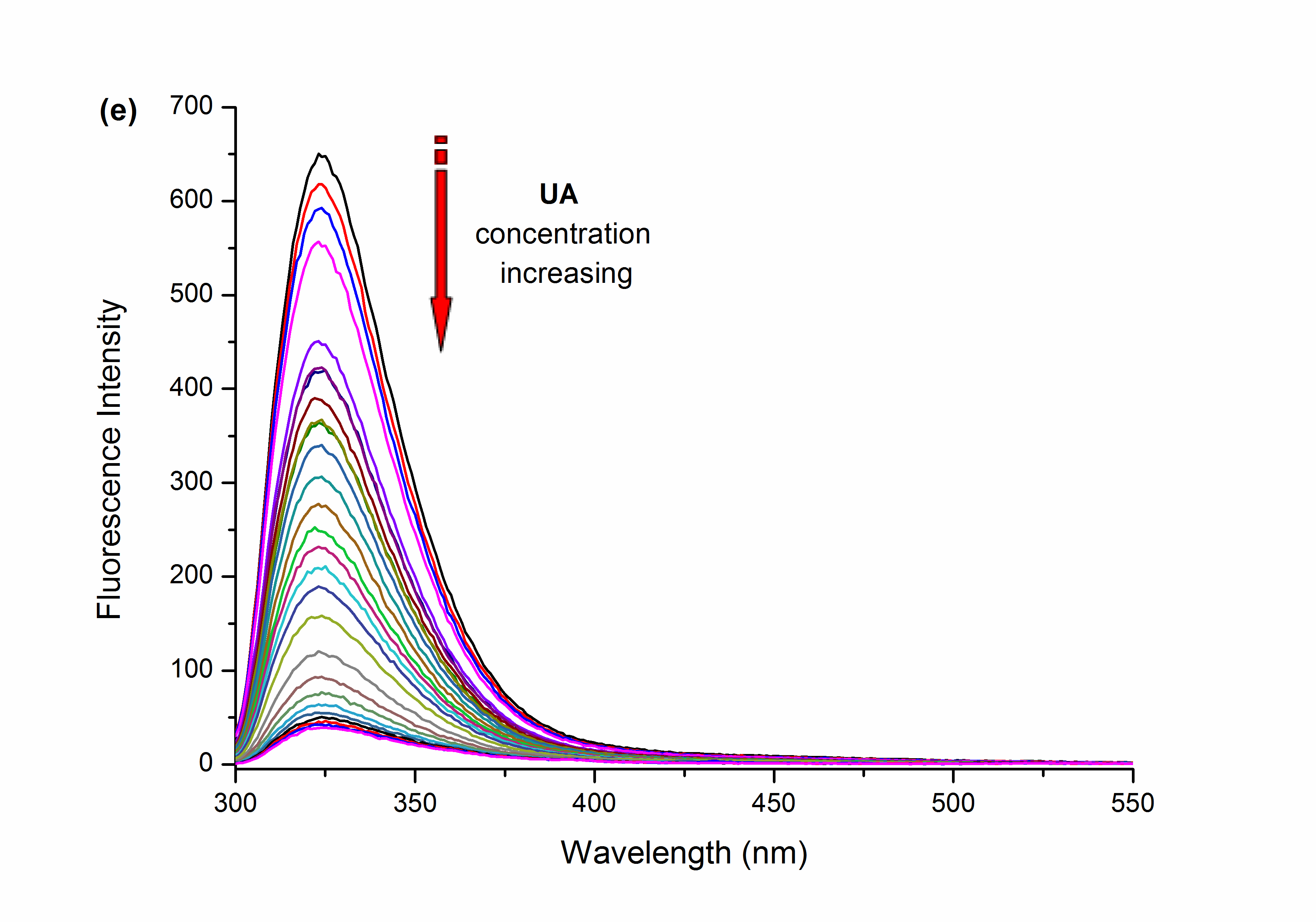


**Supplementary Figure 3.** (a-j) Fluorescence spectra of **CP6** (1.0 × 10^–5^ M) upon addition of substrates (**A, G, X, HX, UA**) (0-5.43 × 10^–4^ M) in aqueous solution (excited at 290 nm) at room temperature, respectively.


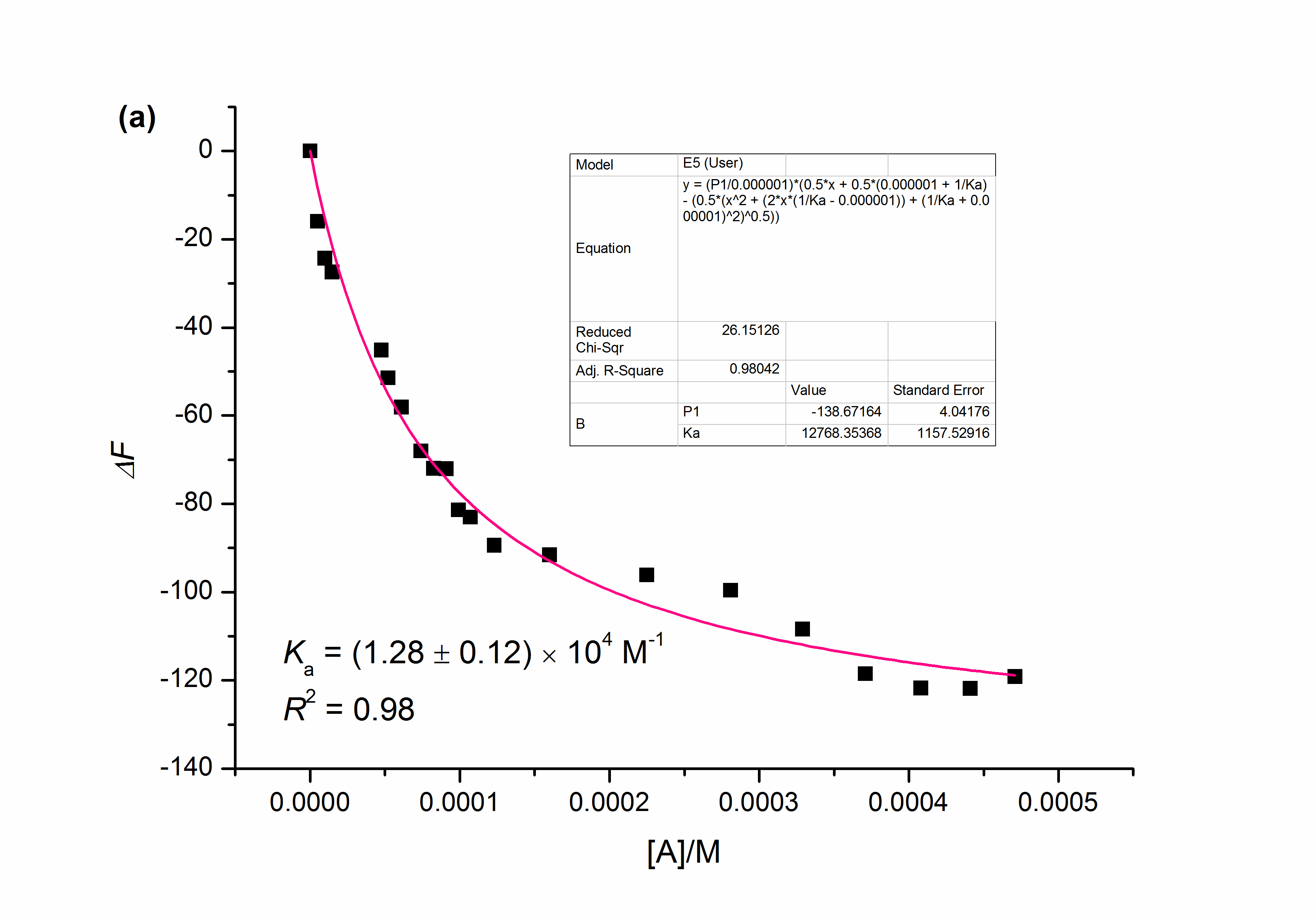

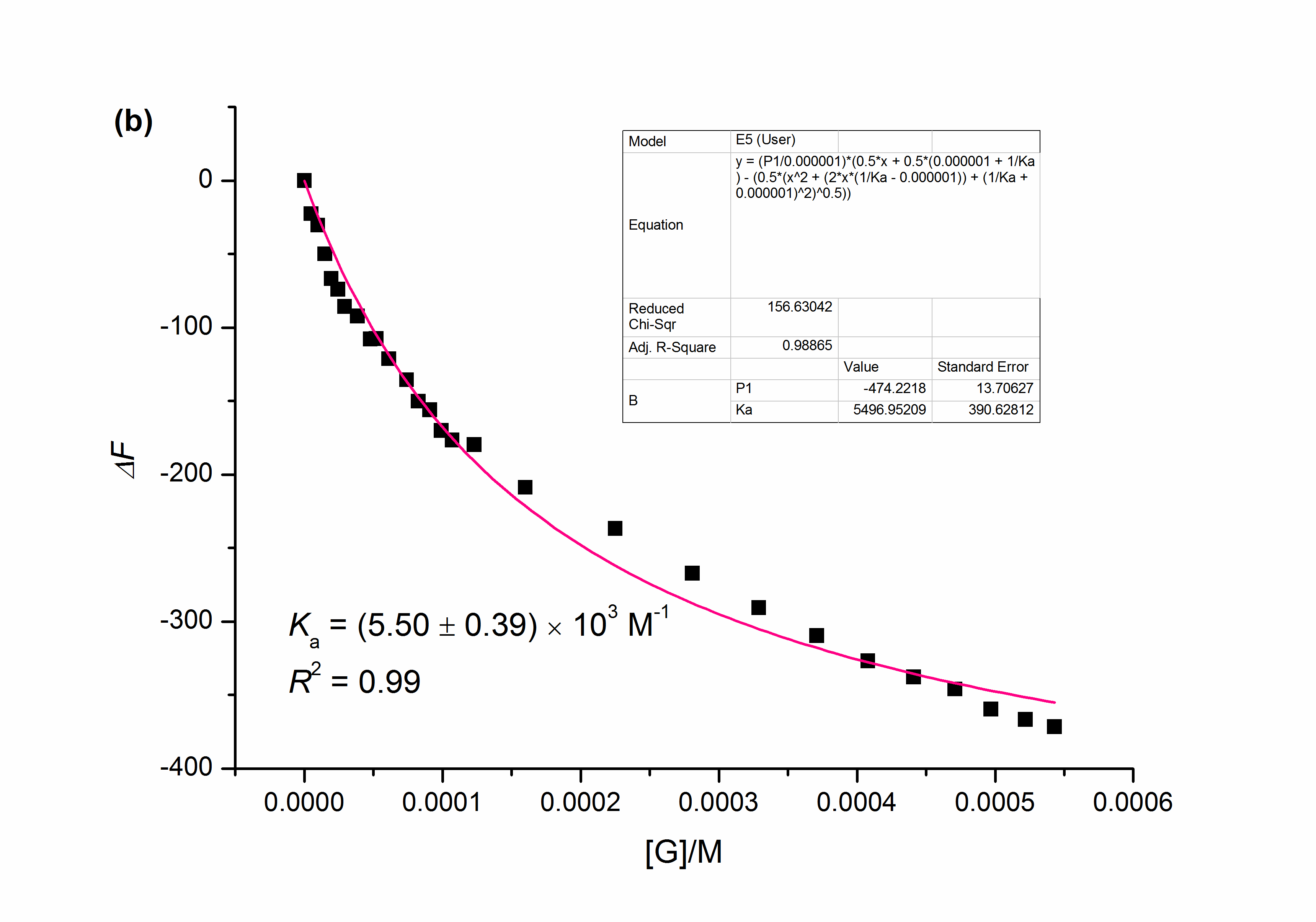


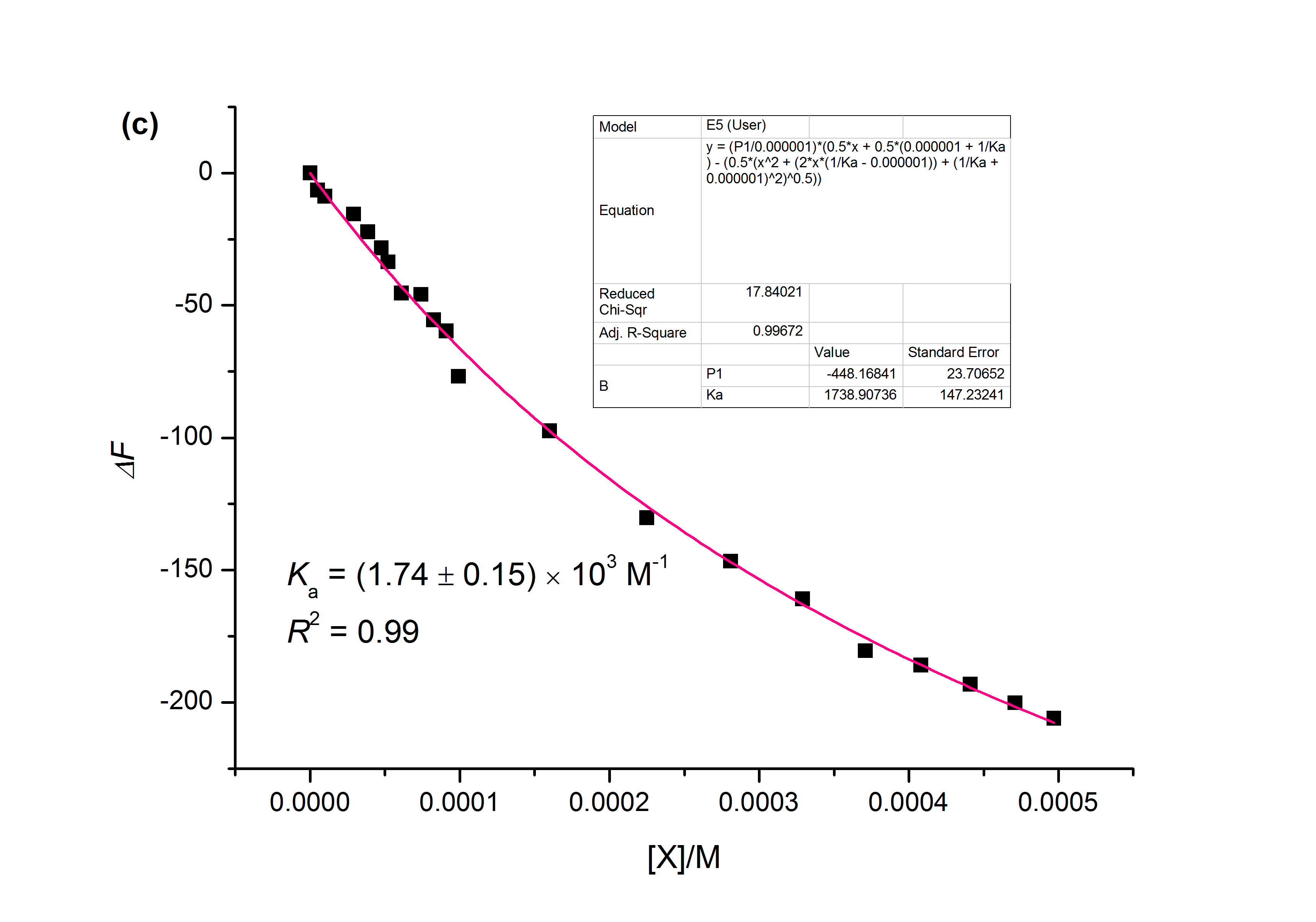

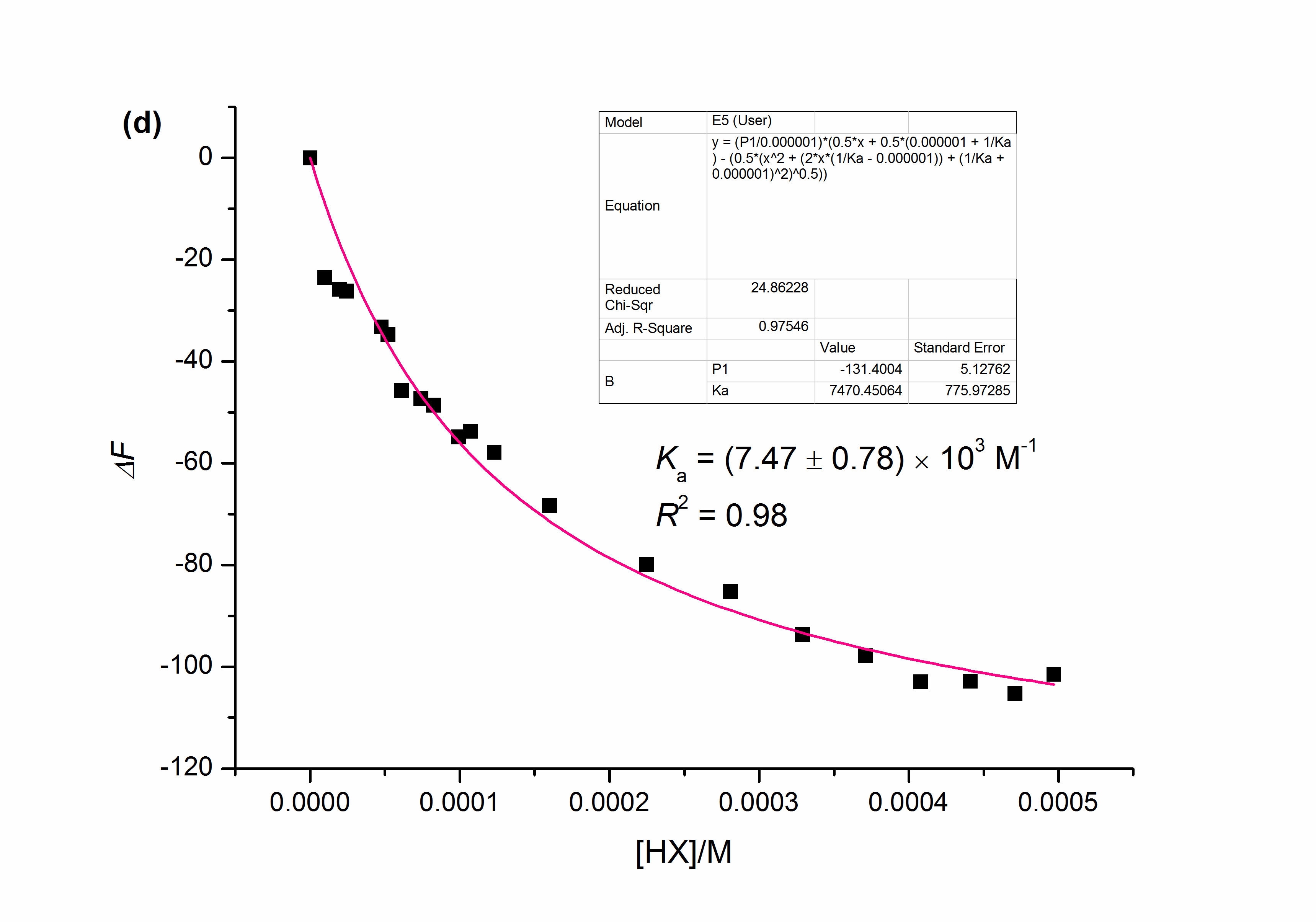


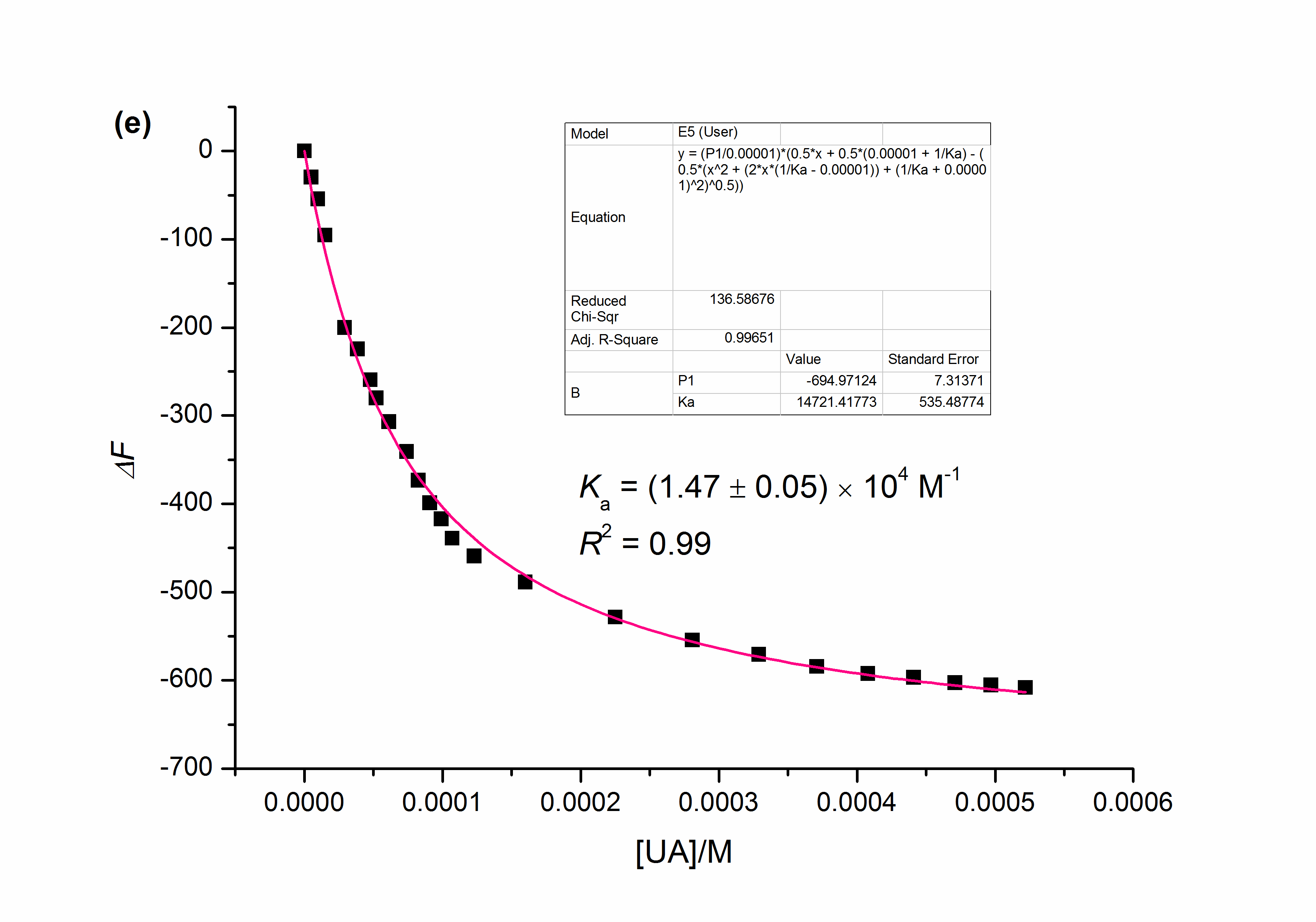


**Supplementary Figure 4.** The fluorescence intensity changes of **CP6** upon addition of substrates (**A, G, X, HX, UA**), respectively. The red solid line was obtained from the non-linear curve-fitting using eq. 1.

**Supplementary Table 1*.*** The association constants between substrates (**A, G, X, HX, UA**) and **CP6** by fluorescence titration experiments.

| Host (guest) | *K*_a_ (L⋅mol^-1^) | Host (guest) | *K*_a_ (L⋅mol^-1^) |
| --- | --- | --- | --- |
| CP6 (A) | (1.28 ± 0.12) × 10^4^ | CP6 (G) | (5.50 ± 0.39) × 10^3^ |
| CP6 (X) | (1.74 ± 0.15) × 10^3^ | CP6 (HX) | (7.47 ± 0.78) × 10^3^ |
| CP6 (UA) | (1.47 ± 0.05) × 10^4^ |  |  |

*References:*

- S1. (a) K. A. Connors, Binding Constants, Wiley: New York, 1987. (b) P. S. Corbin, Ph.D. *Dissertation*, University of Illinois at Urbana-Champaign, Urbana, IL, 1999. (c) P. R. Ashton, R. Ballardini, V. Balzani, M. Belohradsky, M. T. Gandolfi, D. Philp, L. Prodi, F. M. Raymo, M. V. Reddington, N. Spencer, J. F. Stoddart, M. Venturi, D. J. Williams, *J. Am. Chem. Soc.* 118 (1996) 4931−4951. (d) J. Zhang, F. Huang, N. Li, H. Wang, H. W. Gibson, P. Gantzel, A. L. Rheingold, *J. Org. Chem.* 72 (2007) 8935−8938.
